# Efficacy, Safety, and Tolerability of Ansofaxine (LY03005) Extended-Release Tablet for Major Depressive Disorder: A Randomized, Double-Blind, Placebo-Controlled, Dose-Finding, Phase 2 Clinical Trial

**DOI:** 10.1093/ijnp/pyab074

**Published:** 2021-11-08

**Authors:** Weifeng Mi, Fude Yang, Huafang Li, Xiufeng Xu, Lehua Li, Qingrong Tan, Guoqiang Wang, Kerang Zhang, Feng Tian, Jiong Luo, Jielai Xia, Kai Yuan, Lin Lu, Jiahui Deng, Jingwei Tian, Hongyan Zhang

**Affiliations:** 1 Peking University Sixth Hospital, Peking University Institute of Mental Health, NHC Key Laboratory of Mental Health (Peking University), National Clinical Research Center for Mental Disorders (Peking University Sixth Hospital), Beijing, China; 2 Beijing Huilongguan Hospital, Beijing, China; 3 Shanghai Mental Health Center, Shanghai, China; 4 First Affiliated Hospital of Kunming Medical University, Kunming, China; 5 Second Xiangya Hospital of Central South University, Changsha, China; 6 First Affiliated Hospital of the Fourth Military Medical University (Air Force Medical University), Xi’an, China; 7 Wuxi Mental Health Center, Wuxi, China; 8 First Hospital of Shanxi Medical University, Taiyuan, China; 9 Second Hospital of Shanxi Medical University, Taiyuan, China; 10 Beijing Anding Hospital of Capital Medical University, Beijing, China; 11 Fourth Military Medical University of Chinese People’s Liberation Army, Statistical Analysis Teaching and Research Section, Xi’an, China; 12 Yantai University, Yantai, China

**Keywords:** Ansofaxine, major depressive disorder, Phase 2 Clinical Trial

## Abstract

**Background:**

Ansofaxine (LY03005) extended-release tablet is a potential triple reuptake inhibitor of serotonin, norepinephrine, and dopamine. This study assessed the efficacy, safety, and appropriate dosage of ansofaxine for the treatment of major depressive disorder (MDD).

**Methods:**

A multicenter, randomized, double-blind, placebo-controlled, dose-finding, Phase 2 clinical trial was conducted in China. Eligible patients with MDD (18–65 years) were randomly assigned to receive fixed-dose ansofaxine extended-release tablets (40, 80, 120, or 160 mg/d) or placebo for 6 weeks. The primary outcome measure was a change in the total score on the 17-item Hamilton Depression Rating Scale from baseline to week 6.

**Results:**

A total of 260 patients were recruited from October 2015 to September 2017, and 255 patients received the study drug as follows: 40 mg (n = 52), 80 mg (n = 52), 120 mg (n = 51), and 160 mg (n = 51) ansofaxine and placebo (n = 49). Significant differences were found in mean changes in 17-item Hamilton Depression Rating Scale total scores at week 6 in the 4 ansofaxine groups vs placebo (−12.46; *χ*^*2* ^=^ ^−9.71, *P* = .0447). All doses of ansofaxine were generally well-tolerated. Treatment-related adverse events occurred in 141 patients (303 cases), yielding incidence rates of 51.92%, 65.38%, 56.86%, and 62.75% in the 40-, 80-, 120-, and 160-mg ansofaxine groups and 38.78% in the placebo group.

**Conclusion:**

Active doses (40, 80, 120, and 160 mg/d) of ansofaxine in a controlled setting were safe, tolerated, and effective in improving depression symptoms in MDD patients.

**Significance Statement**

The global burden of depression is so heavy that the development of new antidepressant drugs with more powerful antidepressant effects and fewer side effects is still a top priority. We performed a Phase 2 clinical trial to explore the safety, efficacy, and tolerability of ansofaxine extended-release (ER) tablet for the treatment of MDD. This was a multicenter, randomized, double-blind, placebo-controlled, dose-finding trial in which patients received 6 weeks of treatment with ansofaxine ER tablets at doses of 40, 80, 120, or 160 mg/d or placebo. The results showed that 4 doses of ansofaxine ER tablet exerted antidepressant effects and were safe and well-tolerated. Triple reuptake inhibitors block the reuptake of serotonin, norepinephrine, and dopamine from the synapse, with a stronger effect on dopamine reuptake. The results of this trial support further evaluations of ansofaxine ER tablet as a new drug for the treatment of patients with MDD.

## Introduction

Major depressive disorder (MDD) is a common illness that severely limits psychosocial functioning and diminishes quality of life. In 2008, the World Health Organization ranked MDD as the third leading cause of disease burden worldwide and projected that MDD will rank first by 2030. The 12-month prevalence of MDD varies considerably among countries but is approximately 6% overall (Kessler and Bromet, 2013). In China, depression is also one of the most common mental illnesses, with a 12-month prevalence of 3.6% and lifetime prevalence of 6.8% (Huang et al., 2019). For the treatment of depression, antidepressants that affect monoamine systems (dopamine [DA], serotonin [5-hydroxytryptamine (5-HT)], and norepinephrine [NE]) are primary or first-line medications, including tricyclic antidepressants, monoamine oxidase inhibitors, selective serotonin reuptake inhibitors (SSRIs), serotonin and NE reuptake inhibitors (SNRIs), and serotonin receptor partial agonist/reuptake inhibitors (Ashton et al., 2019). However, these drugs are characterized by a slow onset and may have some side effects. Triple reuptake inhibitors (TRIs) block the reuptake of 5-HT, NE, and DA from the synapse, with stronger efficacy on DA reuptake. They may have the additional effect of enhancing neurotransmission of all 3 monoamine systems and potential advantages of a rapid onset of action, amelioration of symptoms of anhedonia and sexual dysfunction, and improvements in cognitive function, reward-motivated function, and goal-oriented behavior (Tran et al., 2012).

Ansofaxine hydrochloride ([±]-4-[2-(dimethylamino)-1-(1-hydroxycyclohexyl) ethyl] phenyl 4-methylbenzoate hydrochloride dihydrate [LY03005, LPM570065]; see supplementary Figure 1; https://pubchem.ncbi.nlm.nih.gov/compound/56955395#section=Information-Sources) is a new chemical compound that is formulated as ansofaxine extended-release (ER) oral tablets for the treatment of adults with MDD. Ansofaxine has a high affinity for the DA transporter, NE transporter, and serotonin transporter and significantly inhibits the reuptake of DA, NE, and 5-HT, making it a potential TRI for the treatment of MDD (Zhang et al., 2014). Microdialysis studies showed that ansofaxine hydrochloride inhibited the reuptake of DA, NE, and 5-HT and increased extracellular 5-HT, DA, and NE levels in the rat striatum after acute and chronic administration (Zhang et al., 2014). The ability of ansofaxine to increase extracellular 5-HT and NE levels in the rat striatum is similar to desvenlafaxine, but its ability to increase extracellular DA levels is approximately 2 to 3 times stronger than desvenlafaxine. Although ansofaxine is a methyl benzoate of desvenlafaxine and can be quickly converted to desvenlafaxine in vivo, ansofaxine and desvenlafaxine can coexist in the blood and brain because of the liposolubility of ansofaxine, with similar concentrations of ansofaxine and desvenlafaxine in the brain when equivalent oral doses of the 2 drugs are given (Zhang et al., 2014; Guo et al., 2018; Ashton et al., 2019). Additionally, ansofaxine is the most balanced TRI that has been studied to date in clinical trials, in which the reuptake of DA, NE, and 5-HT was strongly inhibited. DA plays an important role in the pathogenesis of depression (Nestler and Carlezon, 2006; Grace, 2016). The mesocorticolimbic DA system has been linked to rewarding events and incentive-driven behaviors, and low dopaminergic activity may lead to loss of interest and anhedonia, a core symptom of depression (Nestler and Carlezon, 2006). Studies have shown that a rapid increase in DA levels in the synaptic cleft in the midbrain-cortical-marginal pathway can ameliorate the delay in the treatment of depression, whereas an increase in DA levels in the hypothalamus can enhance dopaminergic nerve stimulation of the nucleus accumbens, thereby improving pleasure, cognition, and sexual dysfunction and rewarding incentive-driven and goal-oriented behaviors.

Preclinical safety studies confirmed that ansofaxine hydrochloride has good safety and tolerability properties (Zhang et al., 2014; Li et al., 2018). Ansofaxine ER tablet has also completed 3 Phase 1 studies in health volunteers: a single ascending-dose study, a diet effect study, and a multiple ascending-dose study (registered at http://www.chinadrugtrials.org.cn/index.html; nos. CTR20130364, CTR20140333, and CTR20140418). The results of these Phase 1 studies showed that the pharmacokinetics of ansofaxine ER tablet have a dose-proportional relationship in the range of 20 to 200 mg/d and that diet had no significant effect on its pharmacokinetics. The results also indicated that ansofaxine ER tablet was safe and well-tolerated in the range of 20 to 200 mg/d in the single ascending-dose study or 40–160 mg/d in the multiple ascending-dose study. The main adverse reactions included nausea, vomiting, diarrhea, dizziness, and elevated total bilirubin or alanine aminotransferase. The full results, however, have not yet been published. In response to the Phase 1 study results, ansofaxine ER tablet was approved for Phase 2 clinical trials. Thus, to investigate the clinical effect of ansofaxine ER tablet, the present study (ClinicalTrial.gov; no. NCT03785652) was designed to explore the optimal dose range of ansofaxine ER tablet and preliminarily assess its safety, efficacy, and tolerability in patients with MDD.

## METHODS

### Study Design

This multicenter, randomized, double-blind, placebo-controlled, parallel-group, dose-finding, Phase 2 study was conducted at 10 hospitals in China (the hospitals are listed in “Additional Contributions” below) from October 2015 to September 2017. The protocol received independent ethics committee approval from the 10 hospitals before the trial began. The study was conducted in accordance with appropriate laws and regulations, including Good Clinical Practice and the Declaration of Helsinki. Written informed consent was obtained from all patients before screening. All researchers from the 10 hospitals underwent protocol consistency training, and all raters were given scale-rating consistency training to ensure consistency among hospitals in this multicenter trial.

### Patients

#### Inclusion Criteria

**—**Individuals 18 to 65 years old with a diagnosis of MDD based on Diagnostic and Statistical Manual of Mental Disorders, 4th edition, text revision criteria who experienced a single episode or recurrent episodes without psychotic symptoms were included. At screening and baseline (day 0), total scores for eligibility were ≥20 on the 17-item Hamilton Depression Rating Scale (HAMD_17_), ≥2 on HAMD_17_ item 1 (depressive mood), and ≥4 on the Clinical Global Impressions-Severity (CGI-S) scale (moderately ill).

#### Exclusion Criteria

—Individuals who were resistant to venlafaxine and antidepressant treatment, had significant risk of suicide (HAMD_17_ item 3 score [suicide item] ≥3), significant placebo response (≥25% decrease in HAMD_17_ total score during the washout period from screening to baseline), another psychiatric diagnosis, unstable physical disease at screening, clinical abnormalities on physical examination, electrocardiogram (ECG), laboratory tests, or urine drug screen at screening, systematic psychotherapy (interpersonal relationship therapy, dynamic therapy, and cognitive behavioral therapy), or transcranial magnetic stimulation within 3 months prior to screening and light therapy within 2 weeks prior to screening (see supplementary Protocol) were excluded.

### Randomization and Masking

Patients were randomly assigned to placebo or 1 of 4 fixed-dose ansofaxine ER tablets (40, 80, 120, or 160 mg/d) in a 1:1:1:1:1 manner using a web-based dynamic randomization system that generated unique container numbers instituted by an external independent third party. A randomized block schedule was used for the drug randomization lists. The sample size was designed to be 260 (52 per group). Patients, clinicians, and independent outcome raters were masked to treatment allocation until study completion (see supplementary Protocol).

### Procedures

This trial comprised 3 periods: screening period of up to 1 week, placebo washout period of 1 week, and a double-blind treatment period of 6 weeks. In the screening period, the patients underwent physical and psychiatric examinations by researchers and laboratory and ECG examinations. After screening, MDD patients who met the inclusion criteria entered into the placebo washout period, during which they took 2 placebo tablets orally once daily in the morning for 7 consecutive days. Patients who still met the inclusion criteria after the placebo washout period were randomly assigned to placebo or 1 of 4 fixed-dose ansofaxine ER tablets. The last day of the placebo washout period was also the first day of the double-blind treatment period and was set as baseline (day 0). Participants returned for follow-up visits on study days 7, 14, 28, and 42 (see supplementary Protocol).

### Outcomes

#### Primary Efficacy Measure

**—**The HAMD_17_ was applied at screening and baseline and during the double-blind treatment period on days 7, 14, 28, and 42 (or at the end of weeks 1, 2, 4, and 6). The primary efficacy endpoint was the change in HAMD_17_ total score from baseline to the end of week 6 or the final on-therapy evaluation.

#### Secondary Efficacy Measure

**—**Changes in Hamilton Anxiety Rating Scale (HAMA) total scores, Clinical Global Impressions-Improvement (CGI-I) scores, CGI-S scores, HAMD_17_ factor scores, and Visual Analog Scale of Pain Intensity (VAS-PI) scores were determined. Response rates, with a response defined as a ≥50% decrease in HAMD_17_ total scores from baseline to the final on-therapy evaluation, were also calculated. Finally, remission rates, defined as HAMD_17_ scores ≤7, were determined.

#### Safety and Tolerability Evaluations

**—**Adverse events (AEs), withdrawal caused by AEs, concomitant medications, weight, vital signs, physical examinations (at screening and on completion) at each visit, laboratory tests, and ECGs were determined (see supplementary Protocol).

### Statistical Analysis

The data were analyzed using SAS 9.2 software (SAS Institute, Cary, NC, USA). The efficacy analysis was mainly based on the full-analysis set (FAS), which was defined as all randomized patients who received at least 1 dose of the study drug during the double-blind treatment period and had both baseline and at least 1 post-baseline measurement of primary efficacy. The FAS used last-observation carried forward imputation.

Changes in HAMD_17_ total scores from baseline were analyzed using ANCOVA, with treatment and study site as fixed factors and HAMD_17_ total score at baseline as the covariate. The least-squares mean, together with the 95% or 90% confidence interval (CI), was estimated for each treatment group and for differences between groups, respectively. The secondary endpoints included HAMA scores, CGI-S scores, HAMD_17_ factor scores, and VAS-PI scores, which were analyzed the same way as the primary endpoint but with replacement of the corresponding baseline with the respective baseline value. The other secondary endpoints included CGI-I scores, response rates, and remission rates, which were analyzed using the Cochran-Mantel-Haenszel *χ*² test with adjustment for the study site factor.

Safety analyses were based on the safety population (safety set), which included all randomized patients who received at least 1 dose of the study drug during the double-blind treatment period. Listings and summary tabulations of AEs were reported. AEs were classified according to Medical Dictionary for Regulatory Activities terminology.

This was a dose-finding study, and all statistical tests were conducted using 2-sided tests with 10% type 1 error rates unless otherwise stated. Values of *P *< .1 were considered statistically significant.

### RESULTS

**Figure 1.**
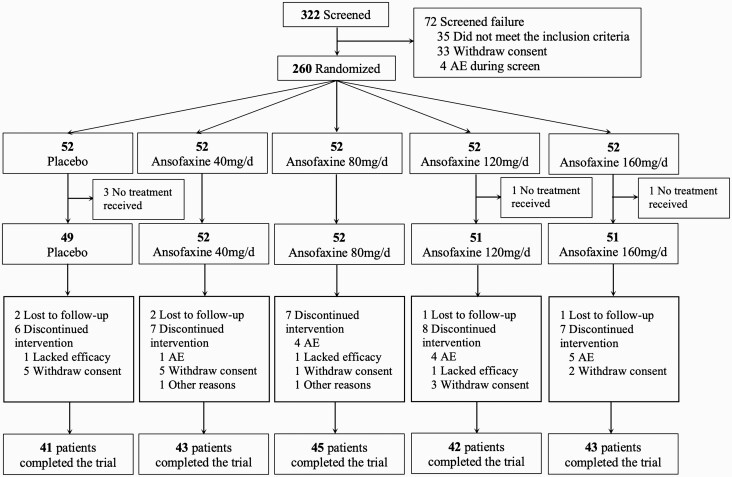
Study design and disposition of patients. AE, adverse event.

We screened 332 patients for this trial. Screening failure occurred in 72 patients, and 260 patients were randomized. Five patients did not receive the drug treatment, and 255 patients received the study drug (safety set) as follows: placebo (n = 49) and ansofaxine ER tablets at doses of 40 mg/d (n = 52), 80 mg/d (n = 52), 120 mg/d (n = 51), or 160 mg/d (n = 51). During the double-blind treatment period, 17.69% of the patients (n = 46, 46/260) withdrew from the study. The reasons for discontinuation are shown in Figure 1. Withdrawal rates did not significantly differ among the 5 groups (*P* = .9017). Nine of the randomized patients did not have at least 1 post-baseline measurement of primary efficacy; thus, the FAS included 246 patients: 49 patients in the placebo group, 49 patients in the 40-mg/d ansofaxine group, 50 patients in the 80-mg/d ansofaxine group, 50 patients in the 120-mg/d ansofaxine group, and 48 patients in the 160-mg/d ansofaxine group. Table 1 shows the demographic and baseline characteristics in the FAS set. There were no significant differences among the treatment groups (all *P *> .1).

**Table 1.** Demographic and Baseline Characteristics (FAS)

		Placebo (n = 49)	Ansofaxine ER tablets				P
			40 mg (n = 49)	80 mg (n = 50)	120 mg (n = 50)	160 mg (n = 48)	
Age (y), mean (SD)		34.93 (11.54)	35.29 (11.44	34.38 (11.57)	33.75 (11.63)	35.14 (12.16)	.9650^*a*^
Sex, no. (%)	Male	18 (36.73)	20 (40.82)	19 (38.00)	19 (38.00)	14 (29.17)	.8082^*b*^
	Female	31 (63.27)	29 (59.18)	31 (62.00)	31 (62.00)	34 (70.83)	
Han ethnicity, no. (%)		47 (95.92)	48 (97.96)	4 8(96.00)	50 (100.0)	45 (93.75)	.4815^*a*^
Weight (kg), mean (SD)		61.73 (11.02)	57.97 (8.59)	59.94 (10.97)	61.11 (12.27)	56.91 (10.22)	.1315^*a*^
BMI (kg/m^2^), mean (SD)		22.56 (3.27)	21.52 (2.62)	22.33 (3.44)	22.03 (3.07)	21.11 (2.96)	.1322^*a*^
Length of current MDE (mo), mean (SD)		10.49 (13.82)	8.33 (7.37)	12.69 (25.96)	9.02 (10.98)	8.78 (13.86)	.5905^*a*^
HAMD_17_ score, mean (SD)		23.63 (3.40)	22.69 (2.55)	23.18 (2.54)	23.68 (3.00)	23.96 (3.05)	.2308^*a*^
CGI-S score, mean (SD)		4.63 (0.67)	4.51 (0.58)	4.46 (0.54)	4.56 (0.64)	4.60 (0.68)	.6508^*a*^

Abbreviations: BMI, body mass index; FAS, full analysis set; ER, extended-release; HAMD_17_, 17-item Hamilton Depression Rating Scale; CGI-S, Clinical Global Impressions-Severity.

^*a*^ANOVA/eank sum test.

^*b*^χ ^2^ test.

**Figure 2.**
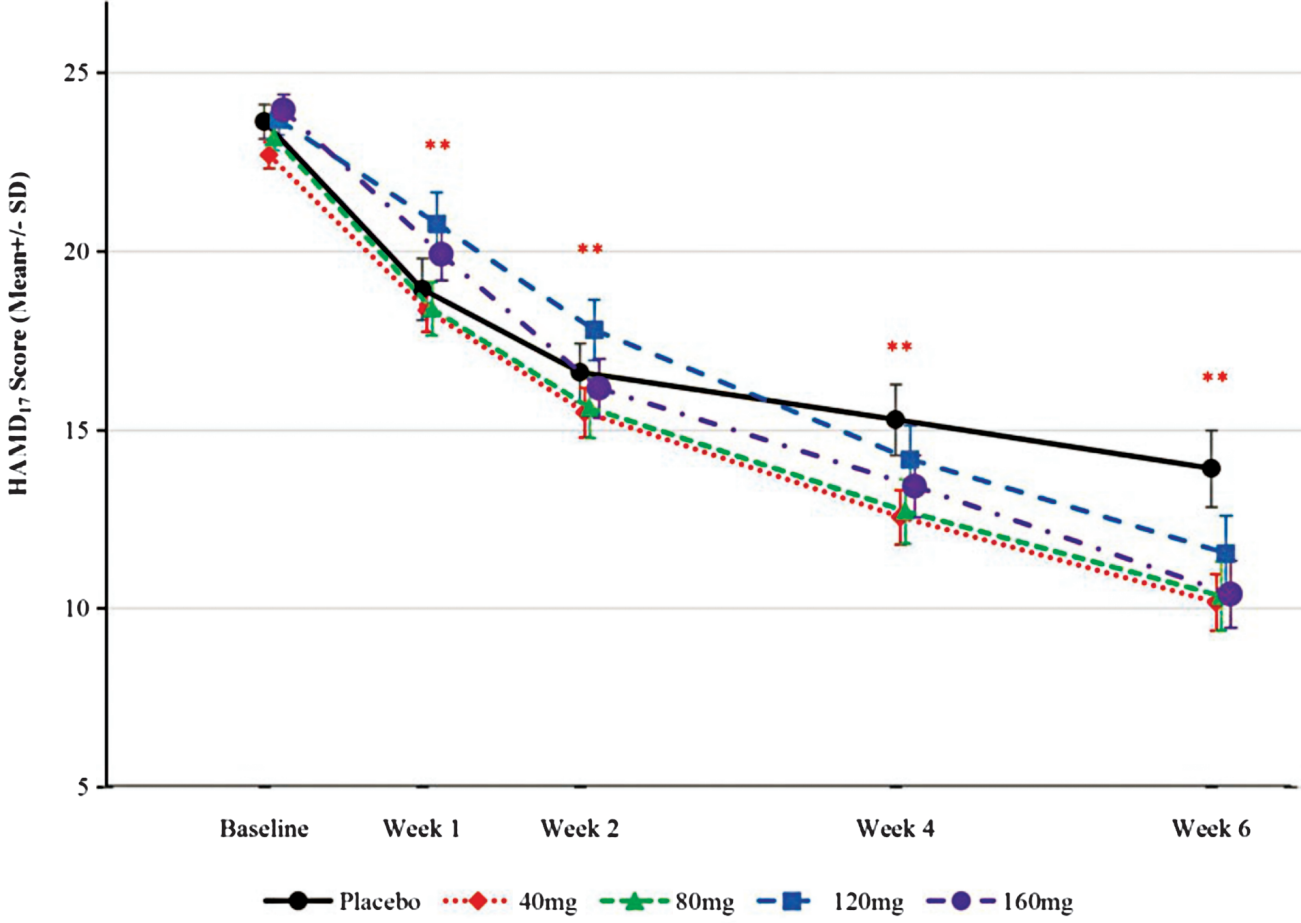
HAMD17 total scores during the double-blind treatment phase (FAS). ^**^P < .01, compared with placebo. FAS, full analysis set; HAMD17, 17-item Hamilton Depression Rating Scale.

Mean changes from baseline in HAMD_17_ total scores at week 6 were −9.71, −12.53, −12.84, −12.14, and −13.56 in the placebo and 40-, 80-, 120-, and 160-mg/d ansofaxine groups, respectively. The differences among the 5 treatment groups were statistically significant (ANCOVA, *χ*^*2*^ = −9.71, *P* = .0447). Adjusted mean differences in HAMD_17_ total scores (95% CI) that were obtained by comparing placebo with the 4 ansofaxine dose groups were statistically significant (−2.92 [−6.09 to 0.24] for the 40-mg group, −3.08 [−6.22 to 0.05] for the 80-mg group, −2.43 [−5.56 to 0.70] for the 120-mg group, and −3.69 [−6.85 to −0.52] for the 160-mg group; Table 2). The 4 groups presented a greater reduction of HAMD_17_ total scores over time compared with the placebo group, and the difference was statistically significant at the end of week 1 (Figure 2).

**Table 2.** Changes of HAMD_17_ Total Scores, HAMA Total Scores, HAMA Somatic Anxiety Factor, and HAMD_17_ Anxiety/Somatization Factor at the End of Week 6 (FAS, LOCF)

	Placebo (n = 49)	Ansofaxine ER tablets				*P*_(a)_ ^*a*^
		40 mg (n = 49)	80 mg (*n* = 50)	120 mg (n = 50)	160 mg (n = 48)	
HAMD_17_						
LSMEAN	−9.54	−12.46	−12.62	−11.97	−13.23	.0447
Average difference from placebo group^*b*^ (90% CI)		−2.92 (−5.05, −0.80)	−3.08 (−5.19, −0.98)	−2.43 (−4.53, −0.33)	−3.69 (−5.82, −1.56)	
Average difference from placebo group^*b*^ (95% CI)		−2.92 (−6.09, 0.24)	−3.08 (−6.22, 0.05)	−2.43 (−5.56, 0.70)	−3.69 (−6.85, −0.52)	
HAMA total scores						
LSMEAN	−6.99	−9.43	−10.24	−8.80	−10.31	.0329
Average difference from placebo group (90% CI)		−2.44 (−4.39, −0.49)	−3.24 (−5.17, −1.31)	−1.81 (−3.74, 0.12)	−1.81 (−3.74, 0.12)	
HAMA somatic anxiety factor						
LSMEAN	−2.20	−3.44	−3.80	−2.88	−3.74	.0295
Average difference from placebo group (90% CI)		−1.23 (−2.18, −0.29)	−1.59 (−2.54, −0.65)	−0.68 (−1.62, 0.27)	−1.53 (−2.49, −0.58)	
HAMD_17_ anxiety/somatization factor						
LSMEAN	−2.50	−3.35	−3.56	−3.22	−3.68	.0724
Average difference from placebo group (90% CI)		−0.85 (−1.59, −0.12)	−1.06 (−1.79, −0.33)	−0.72 (−1.45, 0.01)	−1.18 (−1.92, −0.45)	

Abbreviations: CI, confidence interval; ER, extended-release; FAS, full analysis set; HAMD_17_, 17-item Hamilton Depression Rating Scale; LOCF, last observation carried forward; LSMEAN, least square mean.

^*a*^ *P* value (a) by regression of ANCOVA for comparisons across study groups.

^*b*^Average differences and 90% CIs were obtained from the regression model (least-square approach) after adjusting other model factors.

At the end of week 6, mean changes in HAMA total scores, the HAMA somatic anxiety factor, and the HAMD_17_ anxiety/somatization factor were significantly higher for 3 of the ansofaxine groups (40, 80, and 160 mg/d) but not for the 120-mg ansofaxine group (*P* < .1; Table 2) compared with placebo.

**Figure 3.**
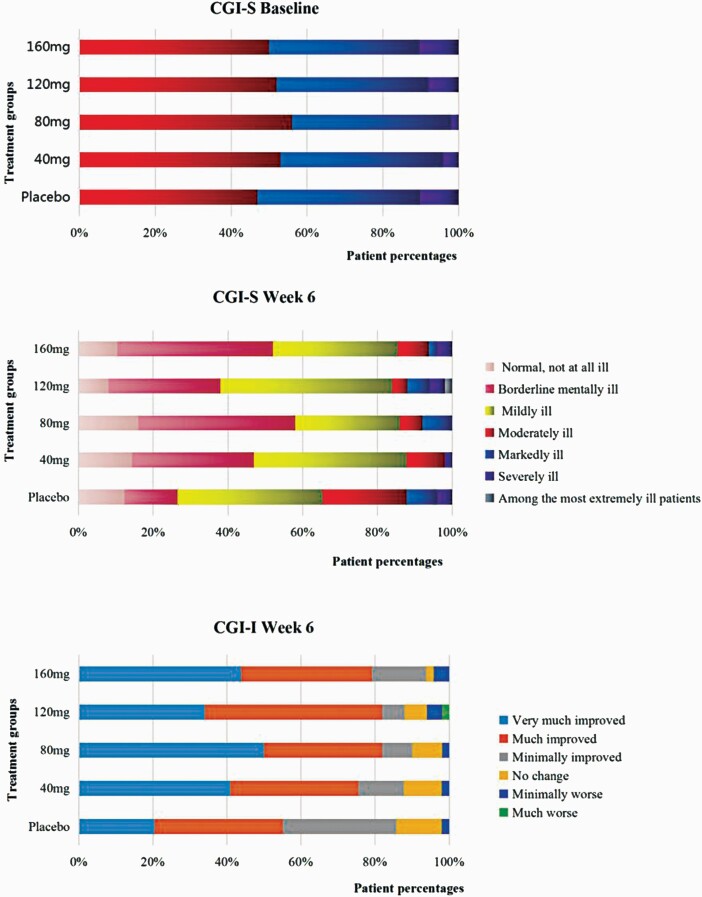
CGI-S and CGI-I scores at baseline and the end of week 6 (FAS). FAS, full analysis set; CGI-I, Clinical Global Impressions-Improvement; CGI-S, Clinical Global Impressions-Severity.

CGI-I scores were significantly higher in the 4 ansofaxine groups than in the placebo group (all *P* < .1). The total number and proportion of “normal, borderline mentally ill, and mildly ill” participants, assessed by the CGI-S at the end of week 6, were significantly higher compared with the placebo group (Figure 3).

At the end of week 6, there was no significant difference in VAS-PI scores (total pain, headache, back pain, joint pain, abdominal pain, or others) among the 5 groups (*P *> .1). Response rates, based on the HAMD_17_, were higher in the 40-, 80-, 120-, and 160-mg/d ansofaxine groups than in the placebo group (FAS, *P* < .05; supplementary Table 1; supplementary Figure 2).

A total of 174 patients (among a total of 457 cases) experienced treatment-emergent AEs (TEAEs) based on the investigators’ judgment: 30 (61.22%) in the placebo group, 34 (65.38%) in the 40-mg ansofaxine group, 39 (75.00%) in the 80-mg ansofaxine group, 36 (70.59%) in the 120-mg ansofaxine group, and 35 (68.63%) in the 160-mg ansofaxine group. No significant differences were found in the incidence of TEAEs among groups (*P* = .6423). In terms of severity, 119 patients (46.67%) had mild TEAEs, 43 (16.86%) had moderate TEAEs, and 12 (4.71%) had severe TEAEs.

A total of 141 participants (303 cases) experienced AEs that might be associated with the study drug (TRAEs) based on the investigators’ judgment. Incidence rates of TRAEs were 51.92% (27 patients, 65 cases), 65.38% (34 patients, 68 cases), 56.86% (29 patients, 70 cases), and 62.75% (32 patients, 70 cases) in the 40-, 80-, 120-, and 160-mg/d ansofaxine groups, respectively, and 38.78% (19 patients, 30 cases) in the placebo group. The severity of TRAEs was mild in 103 patients (40.39%), moderate in 30 patients (11.76%), and severe in 8 patients (3.14%).

Fourteen patients withdrew from the double-blind treatment period because of TEAEs, including 1 patient (1.92%) in the 40-mg ansofaxine group, 4 patients (7.69%) in the 80-mg ansofaxine group, 4 patients (7.84%) in the 120-mg ansofaxine group, and 5 patients (9.80%) in the 160-mg ansofaxine group. No patients withdrew in the placebo group. The difference among groups was statistically significant (*P* = .0948). TEAEs that resulted in withdrawal were mainly nausea, headache, and dizziness and judged to be definitely, probably, or possibly related to ansofaxine.

TRAEs with a ≥5% incidence in any dose group and with an incidence at least twice as high as in the placebo group included decreased appetite, chest discomfort, fatigue, lethargy, constipation, nausea, dry mouth, palpitations, and blurred vision. The incidences of nausea and chest discomfort significantly differed among groups (*P* = .0665 and *P* = .0913, respectively). The 3 most common TRAEs were nausea, lethargy, and decreased appetite (Table 3). Notably, throughout the whole trial, only 1 case reported sexual dysfunction in the 80-mg ansofaxine group.

**Table 3.** Adverse Events With an Incidence ≥5% and at Least Twice as High as in the Placebo Group During the Double-Blind Period (SS)

Adverse Event, no. (%)	Placebo (n = 49)	Ansofaxine ER tablets				Fisher *P*^*a*^
		40 mg (n = 52)	80 mg (n = 52)	120 mg (n = 51)	160 mg (n = 51)	
Nausea	3 (6.12)	10 (19.23)	10 (19.23)	11 (21.57)	14 (27·45)	.0665
Decreased appetite	1 (2.04)	3 (5.77)	1 (1.92)	4 (7.84)	0 (0)	.1811
Chest discomfort	0 (0)	0 (0)	1 (1.92)	0 (0)	3 (5·88)	.0913
Fatigue	1 (2.04)	0 (0)	2 (3.85)	2 (3.92)	3 (5·88)	.5342
Lethargy	0 (0)	1 (1.92)	2 (3.85)	5 (9.8)	3 (5.88)	.1278
Constipation	0 (0)	2 (3.85)	2 (3.85)	1 (1.96)	3 (5·88)	.6251
Dry mouth	1 (2.04)	1 (1.92)	3 (5.77)	2 (3.92)	2 (3·92)	.9040
Palpitations	1 (2.04)	0 (0)	2 (3.85)	2 (3.92)	4 (7·84)	.2743
Blurred vision	0 (0)	3 (5.77)	2 (3.85)	0 (0)	3 (5·88)	.1577

Abbreviations: ER, extended-release; no., number; SS, safety set. Treatment drug-related adverse events.

^*a*^Fishers exact test was used for comparisons between groups.

During the study, 2 serious AEs (SAEs) occurred, both in the 40-mg/d ansofaxine group, including hypomania and the exacerbation of depression. Both events were judged to be related to the study drug and had alleviated by the end of the study. No deaths occurred.

Concomitant medications are shown in supplementary Table 2. Ansofaxine ER tablets might increase supine diastolic pressure and orthostatic diastolic pressure (supplementary Tables 3 and 4). Ansofaxine ER tablets did not appear to influence supine or orthostatic pulse, weight, or physical examination. Two patients had abnormal ECGs with clinical significance, as determined by the researcher (1 case in the placebo group and 1 case in the 40-mg/d ansofaxine group). Ansofaxine ER tablets slightly influenced the laboratory tests (supplementary Information).

## Discussion

This trial was a dose-finding study that preliminarily explored the effective dose, efficacy, and safety of ansofaxine ER tablets. Significant differences were observed in mean changes at week 6 in HAMD_17_ total scores in the 4 ansofaxine groups (40, 80, 120, and 160 mg/d) vs placebo in patients with MDD. We also found that all doses of ansofaxine were generally well-tolerated and safe.

The active ingredient of ansofaxine ER tablets is a *p*-hydroxybenzoic acid, *O*-desmethylvenlafaxine (ODV). With oral administration, ODV has a T_max_ of approximately 6 hours and t_1/2_ of approximately 11 hours. It is expected that a steady-state plasma concentration would be reached after 3 doses. ODV is an active metabolite of venlafaxine after first-pass metabolism and has a longer half-life than venlafaxine (Klamerus et al., 1992). Venlafaxine is a 5-HT and NE reuptake inhibitor, with a stronger inhibitory action on 5-HT reuptake and weak affinity for α-adrenergic receptors, DA D_2_ receptors, 5-HT_1A_ and 5-HT_2_ receptors, opioid receptors, and benzodiazepine binding sites (Schweizer et al., 1997). Compared with venlafaxine, ODV is metabolized by CYP2D6 and is a weak inhibitor of CYP3A4, which is less affected by liver function (Lessard et al., 1999; Perry and Cassagnol, 2009; Pae, 2011). Unlike ODV, ansofaxine is a potential TRI that has high affinity for the recombinant human DA transporter, recombinant human NE transporter, and recombinant human 5-HT transporter. Dopamine transporter reuptake inhibition may ameliorate MDD patients’ anhedonia and the decline in positive emotion and improve cognition reward motivation and goal-oriented behavior (Zhang et al., 2014; Carvalho et al., 2016; Guo et al., 2018; Meder et al., 2019). It may be less likely to cause sexual dysfunction (Lane, 2015) and have fewer residual symptoms after patients achieve clinical recovery. Additionally, it may be effective against treatment-resistant depression (Clayton et al., 2014).

In this trial, the results showed ansofaxine ER tablets were superior to placebo in all 4 dose groups, which might provide strong evidence for the mechanism explorations in the future. At the end of week 6, changes in HAMD_17_ scores from baseline (controlled by placebo) in the 80- and 160-mg/d groups were better than −3 (Dunlop et al., 2012; Boulenger et al., 2014), which is considered clinically significant according to National Institute for Health and Care Excellence guidelines (https://www.nice.org.uk/guidance/CG23). The results were consistent with previous studies (Brownstone et al., 2012; Boulenger et al., 2014). The treatment response rates were superior to the placebo group and better than vortioxetine, vilazodone, and levomilnacipran in previous studies, which used Montgomery and Asberg Depression Rating Scale scores as the evaluation criterion (Kennedy et al., 2009; Montgomery et al., 2015; Thase et al., 2016; Kornstein et al., 2018). Our results suggest that ansofaxine ER tablets had a significant and rapid antidepressant effect, which became statistically significant at the end of week 1 compared with placebo. It also had an apparent effect in patients with anxiety symptoms. The mechanism of action of ansofaxine may be related to an increase in the inhibition of DA reuptake, which quickly improves depressive symptoms.

The incidences of TRAEs in the 40-, 80-, 120-, and 160-mg/d ansofaxine groups were 51.92%, 65.38%, 56.86%, and 62.75%, respectively. As previously reported, the incidences of AEs were 75.5% for levomilnacipran (Montgomery et al., 2013), 84% for vilazodone, 55% for vortioxetine (Deardorff and Grossberg, 2014), and 77%–80% for desmethylvenlafaxine succinate sustained-release tablets (Boyer et al., 2008; Liebowitz et al., 2008). A meta-analysis that compared AEs among escitalopram and other SSRIs (i.e., citalopram, fluoxetine, paroxetine, and sertraline) and SNRIs (i.e., venlafaxine and duloxetine) found incidences of 73.3%–73.6%, 78.2%, and 77.4%, respectively. The percentages of withdrawal that was attributable to AEs were 1.92%, 7.69%, 7.84%, and 9.80% in the 40-, 80-, 120-, and 160-mg/d ansofaxine groups in the present study. The percentages of withdrawal attributable to AEs were 6.8% for vortioxetine (Boulenger et al., 2014), 9.4% for levomilnacipran (Montgomery et al., 2013), 5.3%–5.4% for escitalopram, 6.3% for other SSRIs (i.e., citalopram, fluoxetine, paroxetine, and sertraline), and 12.9% for SNRIs (i.e., venlafaxine and duloxetine) (Kennedy et al., 2009). These findings showed that the incidences of AEs and rate of discontinuation attributable to AEs of ansofaxine ER tablets were similar to SSRIs and lower than SNRIs, suggesting that the safety and tolerability of ansofaxine ER tablets were similar to these SSRIs and better than other SNRIs. Most AEs that occurred in the present study were mild to moderate, with no unexpected adverse reactions. The most common AEs that were related to the study drug (>5% incidence and twofold higher incidence vs placebo) were nausea, lethargy, decreased appetite, and dry mouth, which were similar to SSRIs and SNRIs (Kennedy et al., 2009; Montgomery et al., 2013). Two SAEs occurred in the 40-mg/d ansofaxine dose group, namely hypomania (mild) and the exacerbation of depression (severe), which were determined to be related to the study drug and stable during follow-up. Similar SAEs have been reported for desmethylvenlafaxine succinate sustained-release tablets Desvenlafaxine Medical Review(s), 2008. All of the above comparisons suggest that ansofaxine ER tablets are safe for MDD patients.

Sexual dysfunction is common during antidepressant treatment. The prevalence of sexual dysfunction in depressive patients who are treated with antidepressants has been found to be 2 times higher than healthy controls (50% vs 24%) (Angst, 1998). The mechanisms that underlie sexual dysfunction are complex (Montejo et al., 2018). Two studies indicated that 27%–65% of female patients and 26%–57% of male depressed patients experienced either a worsening of preexisting difficulties or the emergence of new sexual difficulties in the early weeks of SSRI or SNRI treatment (Williams et al., 2006, 2010). In the present study, the incidence of sexual dysfunction was quite low relative to previous studies, indicating that ansofaxine may not have unwanted sexual side effects. The reason why ansofaxine is associated with a lower incidence of sexual dysfunction is unclear. A meta-analysis of 58 randomized controlled trials and 5 observational studies found some advantages of bupropion, with significantly lower sexual dysfunction compared with SSRIs, such as escitalopram, fluoxetine, paroxetine, and sertraline, which may be related to the mechanisms of action on NE and DA (Reichenpfader et al., 2014). Ansofaxine is a potential TRI with high affinity for the recombinant human DA transporter, recombinant human NE transporter, and recombinant human 5-HT transporter, which may be a mechanism that underlies the lower occurrence of sexual function–related AEs. Further studies should directly investigate the effects of ansofaxine on sexual dysfunction.

Pharmacological studies of desmethylvenlafaxine suggest that it has pain-relieving effects. Eight weeks of treatment with 100 mg/d desmethylvenlafaxine decreased VAS-PI scores. Although changes in VAS-PI scores that were caused by ansofaxine treatment were higher than in the placebo group, the differences were not statistically significant (Bonci and Hopf, 2005; Guiard et al., 2009). This may be related to the relatively small sample size, the patients’ low VAS-PI scores at baseline, and possibly insufficient drug dosages to elicit an antinociceptive effect in this trial.

One possible limitation of the present study was that the number of MDD patients was small. Further studies with a large sample size are required. The main objective of this trial was to determine the optimal dose of ansofaxine ER tablet and preliminarily assess its safety, efficacy, and tolerability. Another limitation of this study was that we did not explore the TRI effect of the different doses of ansofaxine ER tablet, which should be considered in future studies.

In conclusion, this Phase 2, randomized, placebo-controlled clinical trial of ansofaxine ER tablets (40–160 mg/d) demonstrated its safety, efficacy, and tolerability in patients with MDD. Our findings warrant further studies in Phase 3 clinical trials, including larger, multinational, randomized controlled trials.

## Supplementary Material

pyab074_suppl_Supplementary_Data_S1

pyab074_suppl_Supplementary_Data_S2
