# Supplementary material for: Efficacy, Safety, and Tolerability of Ansofaxine (LY03005) Extended-Release Tablet for Major Depressive Disorder: A Randomized, Double-Blind, Placebo-Controlled, Dose-Finding, Phase 2 Clinical Trial
Source: Int J Neuropsychopharmacol. 2021 Nov 8;25(3):252–60. doi: 10.1093/ijnp/pyab074 (PMC8929756; doi:10.1093/ijnp/pyab074)
Supplement: pyab074_suppl_Supplementary_Data_S1 [file pyab074_suppl_supplementary_data_s1.docx]

**Supplementary Information**

| **Figure S1. Chemical structure of ansofaxine** **hydrochloride.** |
| --- |
| 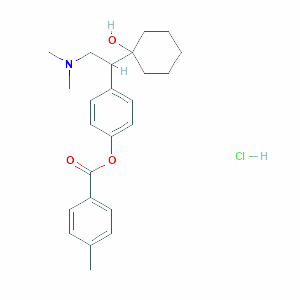 |
| Molecular formula: [C_24_H_32_ClNO_3_](https://pubchem.ncbi.nlm.nih.gov/#query=C24H32ClNO3)  Synonyms: LY03005 hydrochloride or LPM570065 hydrochloride  Source: [https://pubchem.ncbi.nlm.nih.gov/compound/56955395#section = Information-Sources](https://pubchem.ncbi.nlm.nih.gov/compound/56955395#section=Information-Sources) |

| **Figure S2. Response rate and remission rate at the end of week 6 (FAS).** |
| --- |
| *  *  *  *  *  * |
| **p* < 0.05, compared with placebo.  FAS, full analysis set; HAMD_17_, 17-item Hamilton Depression Rating Scale. |

| **Table S1. Response rate and remission rate at the end of week 6 (FAS).** | | | | | | |
| --- | --- | --- | --- | --- | --- | --- |
|  | **Placebo** | **Ansofaxine ER tablets** | | | | *p* _(a)_^1^ |
|  |  | **40 mg** | **80 mg** | **120 mg** | **160 mg** |  |
| **Response Rate (HAMD)** |  |  |  |  |  |  |
| Response, no. (%) | 20 (40.82) | 32 (65.31) | 37 (74.00) | 34 (68.00) | 34 (70.83) | 0.0062 |
| No response, no. (%) | 29 (59.18) | 17 (34.69) | 13 (26.00) | 16 (32.00) | 14 (29.17) |  |
| Total no. | 49 | 49 | 50 | 50 | 48 |  |
| *p* _(b)_^2^ |  | 0.0165 | 0.0008 | 0.0063 | 0.0031 |  |
| **Remission Rate (HAMD)** |  |  |  |  |  |  |
| Remission, no. (%) | 10 (20.41) | 16 (32.65) | 20 (40.00) | 15 (30.00) | 17 (35.42) | 0.3368 |
| No remission, no. (%) | 39 (79.59) | 33 (67.35) | 30 (60.00) | 35 (70.00) | 31 (64.58) |  |
| Total no. | 49 | 49 | 50 | 50 | 48 |  |
| ^1^*p* _(a)_: bidirectional out-of-order CMH-*χ^2^* ^t^est was used for comparisons between groups, controlling central effect.  ^2^*p* _(b)_: controlling central effect, bidirectional out-of-order CMH-*χ^2^* ^t^est was used for comparisons between ansofaxine groups and placebo group.  FAS, full analysis set; ER, extended-release; HAMD_17_, 17-item Hamilton Depression Rating Scale. | | | | | | |

| **Table S2. Concomitant medications during treatment (SS).** | | | | | | | | | | | | | | |
| --- | --- | --- | --- | --- | --- | --- | --- | --- | --- | --- | --- | --- | --- | --- |
| **Items** | **Placebo**  **(*n* = 49)** | | **Ansofaxine ER tablets** | | | | | | | | | | | Fisher *p** |
|  |  |  | **40 mg (*n* = 52)** | | **80 mg (*n* = 52)** | | | **120 mg (*n* = 51)** | | | **160 mg (*n* = 51)** | | |  |
|  | Cases | Patients (%) | Cases | Patients (%) | | Cases | Patients (%) | | Cases | Patients (%) | | Cases | Patients (%) |  |
| Total | 17 | 12(24.49) | 58 | 21(40.38) | | 34 | 18(34.62) | | 44 | 21(41.18) | | 57 | 19(37.25) | 0.4050 |
| Gastrointestinal and metabolic systems | 1 | 1(2.04) | 5 | 4(7.69) | | 5 | 5(9.62) | | 10 | 8(15.69) | | 5 | 4(7.84) | 0.1869 |
| Blood and hematopoietic organs | 0 | 0(0.00) | 0 | 0(0.00) | | 0 | 0(0.00) | | 1 | 1(1.96) | | 1 | 1(1.96) | 0.5889 |
| Cardiovascular system | 2 | 2(4.08) | 1 | 1(1.92) | | 0 | 0(0.00) | | 6 | 3(5.88) | | 2 | 1(1.96) | 0.3680 |
| Genitourinary system and sex hormones | 0 | 0(0.00) | 1 | 1(1.92) | | 0 | 0(0.00) | | 0 | 0(0.00) | | 0 | 0(0.00) | 1.0000 |
| Systematic hormonal use of non sex hormones and insulin | 0 | 0(0.00) | 0 | 0(0.00) | | 1 | 1(1.92) | | 0 | 0(0.00) | | 0 | 0(0.00) | 1.0000 |
| Systemic anti-infective drugs | 1 | 1(2.04) | 7 | 4(7.69) | | 1 | 1(1.92) | | 1 | 1(1.96) | | 4 | 4(7.84) | 0.3659 |
| Antitumor and immunomodulators | 1 | 1(2.04) | 1 | 1(1.92) | | 1 | 1(1.92) | | 0 | 0(0.00) | | 0 | 0(0.00) | 0.8000 |
| Musculoskeletal system | 1 | 1(2.04) | 0 | 0(0.00) | | 2 | 2(3.85) | | 0 | 0(0.00) | | 1 | 1(1.96) | 0.5238 |
| Nervous system | 10 | 6(12.24) | 29 | 17(32.69) | | 18 | 14(26.92) | | 20 | 14(27.45) | | 40 | 14(27.45) | 0.1520 |
| Respiratory system | 0 | 0(0.00) | 1 | 1(1.92) | | 1 | 1(1.92) | | 0 | 0(0.00) | | 0 | 0(0.00) | 1.0000 |
| Others | 1 | 1(2.04) | 13 | 8(15.38) | | 4 | 3(5.77) | | 6 | 6(11.76) | | 3 | 2(3.92) | 0.0775 |
| Non drug therapy | 0 | 0(0.00) | 0 | 0(0.00) | | 1 | 1(1.92) | | 0 | 0(0.00) | | 1 | 1(1.96) | 0.9165 |
| *Fishers Exact Test was used for comparisons between groups.  SS, safety set; ER, extended-release. | | | | | | | | | | | | | | |

| **Table S3. Supine diastolic pressure changes intra- and inter-group at each visit (SS).** | | | | | | | |  |
| --- | --- | --- | --- | --- | --- | --- | --- | --- |
|  | **Placebo**  **(*n* = 49)** | **Ansofaxine ER tablets** | | | | | *p*_(a)_ | |
|  |  | **40 mg (*n* = 52)** | **80 mg (*n* = 52)** | **120 mg (*n* = 51)** | | **160 mg (*n* = 51)** |  |  |
| **Baseline** |  |  |  |  |  | |  | |
| no. (Missing) | 49 (0) | 52 (0) | 52 (0) | 51 (0) | 51 (0) | |  | |
| Mean (SD) | 68.65 (8.58) | 70.94 (8.66) | 69.85 (8.46) | 68.78 (7.02) | 69.27 (8.07) | | 0.6160 | |
| Median | 69 | 71 | 69.5 | 68 | 69 | |  | |
| Min, Max | 48.00, 84.00 | 56.00, 96.00 | 53.00, 83.00 | 56.00, 88.00 | 50.00, 87.00 | |  | |
| **1 week-Baseline** |  |  |  |  |  | |  | |
| no. (Missing) | 48 (1) | 49 (3) | 48 (4) | 47 (4) | 47 (4) | |  | |
| Mean (SD) | 0.88 (5.49) | 1.08 (6.87) | 1.13 (6.20) | 2.85 (6.14) | 2.36 (5.59) | | 0.4028 | |
| Median | 1.5 | 1 | 0.5 | 3 | 1 | |  | |
| Min, Max | -15.00, 13.00 | -13.00, 16.00 | -13.00, 12.00 | -11.00, 16.00 | -10.00, 14.00 | |  | |
| *p* | 0.2752 | 0.2759 | 0.215 | 0.0026* | 0.0057* | |  | |
| **2 week-Baseline** |  |  |  |  |  | |  | |
| no. (Missing) | 44 (5) | 48 (4) | 47 (5) | 43 (8) | 43 (8) | |  | |
| Mean (SD) | 0.36 (8.58) | 0.48 (7.15) | 1.72 (6.86) | 3.30 (7.74) | 3.19 (6.61) | | 0.1723 | |
| Median | 0 | 0 | 1 | 3 | 3 | |  | |
| Min, Max | -20.00, 32.00 | -15.00, 18.00 | -16.00, 17.00 | -12.00, 20.00 | -12.00, 22.00 | |  | |
| *p* | 0.78 | 0.6447 | 0.0916* | 0.0077* | 0.0029* | |  | |
| **4 week-Baseline** |  |  |  |  |  | |  | |
| no. (Missing) | 42 (7) | 44 (8) | 46 (6) | 42 (9) | 43 (8) | |  | |
| Mean (SD) | 0.29 (4.70) | -0.11 (6.57) | 2.46 (7.40) | 2.71 (6.33) | 3.58 (4.97) | | 0.0193* | |
| Median | 0 | -1 | 2 | 2.5 | 3 | |  | |
| Min, Max | -10.00, 10.00 | -12.00, 17.00 | -15.00, 23.00 | -11.00, 15.00 | -6.00, 14.00 | |  | |
| *p* | 0.6955 | 0.9092 | 0.0294* | 0.0082* | < 0.0001* | |  | |
| **6 week-Baseline** |  |  |  |  |  | |  | |
| no. (Missing) | 43 (6) | 47 (5) | 49 (3) | 45 (6) | 46 (5) | |  | |
| Mean (SD) | 1.23 (5.96) | -0.36 (7.91) | 1.76 (6.74) | 2.76 (6.81) | 3.89 (6.10) | | 0.0367* | |
| Median | 0 | 0 | 2 | 3 | 4 | |  | |
| Min, Max | -11.00, 17.00 | -32.00, 15.00 | -14.00, 14.00 | -17.00, 15.00 | -9.00, 18.00 | |  | |
| *p*_(b)_ | 0.1826 | 0.7552 | 0.0745* | 0.0095* | 0.00018 | |  | |
| *p*_(a)_, analysis of variance (ANOVA) was used to compare changes before and after treatment among groups.  *p* _(b)_ , paired *t*-test was used to compare changes before and after treatment in one dosage group.  **p* < 0.1.  SS, safety set; ER, extended-release; SD, standard deviation; no., number. | | | | | | | |  |

| **Table S4. Orthostatic diastolic pressure changes intra- and inter-group at each visit (SS).** | | | | | | |
| --- | --- | --- | --- | --- | --- | --- |
|  | **Placebo**  **(*n* = 49)** | **Ansofaxine ER tablets** | | | | *p* _(a)_ |
|  |  | **40 mg (*n* = 52)** | **80 mg (*n* = 52)** | **120 mg (*n* = 51)** | **160 mg (*n* = 51)** |  |
| **Baseline** |  |  |  |  |  |  |
| no. (Missing) | 49 (0) | 52 (0) | 52 (0) | 51 (0) | 51 (0 ) |  |
| Mean (SD) | 72.96 (9.35) | 73.90 (8.62) | 73.58 (8.71) | 73.06 (7.92) | 72.88 (9.17) | 0.9707 |
| Median | 74 | 73 | 74.5 | 72 | 72 |  |
| Min, Max | 56.00, 92.00 | 60.00, 95.00 | 58.00, 94.00 | 58.00, 92.00 | 56.00, 89.00 |  |
| **1 week-Baseline** |  |  |  |  |  |  |
| no. (Missing) | 48 (1) | 49 (3) | 48 (4) | 47 (4) | 47 (4) |  |
| Mean (SD) | -0.23 (6.21) | 1.80 (7.29) | 1.65 (6.32) | 1.98 (7.08) | 2.23 (7.06) | 0.4100 |
| Median | 0 | 2 | 1 | 1 | 2 |  |
| Min, Max | -18.00, 12.00 | -22.00, 16.00 | -19.00, 18.00 | -13.00, 16.00 | -17.00, 16.00 |  |
| *p* | 0.7994 | 0.0911* | 0.0777* | 0.0617* | 0.0353* |  |
| **2 week-Baseline** |  |  |  |  |  |  |
| no. (Missing) | 44 (5) | 48 (4) | 47 (5) | 43 (8) | 43 (8) |  |
| Mean (SD) | -1.45 (6.14) | 0.48 (6.46) | 2.32 (6.53) | 0.88 (5.61) | 1.77 (7.09) | 0.0584* |
| Median | 0 | 2 | 3 | 0 | 2 |  |
| Min, Max | -20.00, 8.00 | -16.00, 17.00 | -25.00, 14.00 | -9.00, 20.00 | -13.00, 21.00 |  |
| *p* | 0.1234 | 0.6096 | 0.0188* | 0.3073 | 0.1095 |  |
| **4 week-Baseline** |  |  |  |  |  |  |
| no. (Missing) | 42 (7) | 44 (8) | 46 (6) | 42 (9) | 43 (8) |  |
| Mean (SD) | -0.17 (5.96) | 0.16 (7.81) | 2.41 (5.39) | 0.55 (6.32) | 2.91 (6.97) | 0.0999 |
| Median | -0.5 | 1 | 2.5 | 0 | 3 |  |
| Min, Max | -11.00, 13.00 | -26.00, 12.00 | -12.00, 13.00 | -12.00, 21.00 | -12.00, 24.00 |  |
| *p* | 0.8571 | 0.8931 | 0.0039* | 0.5773 | 0.0091* |  |
| **6 week-Baseline** |  |  |  |  |  |  |
| no. (Missing) | 43 (6) | 47 (5) | 49 (3) | 45 (6) | 46 (5) |  |
| Mean (SD) | -0.09 (5.92) | -0.53 (8.38) | 1.98 (6.64) | 1.47 (6.59) | 2.57 (6.61) | 0.1440 |
| Median | 0 | 0 | 2 | 1 | 3 |  |
| Min, Max | -15.00, 14.00 | -27.00, 18.00 | -22.00, 14.00 | -15.00, 17.00 | -11.00, 21.00 |  |
| *p*_(b)_ | 0.9184 | 0.6654 | 0.0422* | 0.1426 | 0.0116 |  |
| *p*_(a)_, analysis of variance (ANOVA) was used to compare changes before and after treatment among groups.  *p*_(b)_ , paired *t*-test was used to compare changes before and after treatment in one dosage group.  **p* < 0.1.  SS, safety set; ER, extended-release; SD, standard deviation; no., number. | | | | | | |

**Safety**

**Concomitant medications**

During treatment, the drug combination is shown in Table S2. There was no significant difference between the four dosage groups (40, 80, 120, or 160 mg/day) of ansofaxine and the placebo group (*p* = 0.4050). The rates of drug combination in the four dosage groups (40, 80, 120, or 160 mg/day) of ansofaxine were 40.38%, 34.62%, 41.18%, and 37.25%, respectively, and the placebo group was 24.49%. The frequencies of combinations of drugs that involved the nervous system was the highest (mainly sedative hypnotics). In the four dosage groups (40, 80, 120, and 160 mg/day) of ansofaxine, these frequencies were 32.69%, 26.92%, 27.45%, and 27.45%, respectively, and the frequency in the placebo group was 12.24%.

**Vital signs**

There was no significant difference in changes in armpit temperature or changes in respiration rate after treatment intra- and inter-group.

**Supine and orthostatic blood pressure**

Ansofaxine ER tablets might increase supine diastolic pressure. From the end of week 1, supine diastolic pressure in the high dosage groups (120 and 160 mg/day) significantly increased (*P* < 0.1). At the end of week 1, supine diastolic pressure in the 80 mg/day group significantly increased (*p* < 0.1). At the end of week 4 and week 6, differences in changes in supine diastolic pressure among the five groups were significant (*p* = 0.0193, *p* = 0.0367). At the end of week 6, the increases in supine diastolic pressure in the placebo group, 40 mg/day group, 80 mg/day group, 120 mg/day group, and 160 mg/day group were 1.23 ± 5.96 mmHg, 0.36 ± 7.91) mmHg, 1.76 ± 6.74 mmHg, 2.76 ± 6.81 mmHg, and 3.89 ± 6.10 mmHg, respectively (see Table S3).

Orthostatic diastolic pressure also increased. At the end of week 2 and week 4, the differences in changes in orthostatic diastolic pressure were statistically significant among the five groups (*p* = 0.584, *p* = 0.0999), but the differences at the end of week 6 were not significant (*p* = 0.144; see Table S4). Supine and orthostatic systolic blood pressure did not significantly change.

**Supine and orthostatic pulse**

Differences in changes in orthostatic pulse among the five groups were statistically significant (*p* = 0.0208) at the end of week 6. Orthostatic pulse change in the placebo group was - (1.70±9.93) times/min, and - (2.43±9.70) times/min, - (2.78±9.33) times/min, (0.93±10.43) times/min, (3.39±12.37) times/min for 80mg/d, 120mg/d and 160mg/d group respectively.

**Physical examination**

Physical examination included lungs, abdomen, spine, limbs, thyroid gland, lymph node, skin mucosa, nervous system, head and neck, facial features, heart, general condition, motor system, and others. During the study period, there was no case who was normal on the baseline physical examination and became abnormal after 6 weeks of treatment.

**Weight**

Differences in changes in weight among the five groups were not significant (*p* > 0.1) at the end of week 6.

**ECG**

During the study period, two patients’ ECGs were abnormal with clinical significance, determined by their researcher. One case in the placebo group had QTc prolongation (extended from 457 MS at baseline to 480 MS at week 6). One case in the 40 mg/day group had an abnormal ECG at the end of week 4 (ECG at baseline was normal but was abnormal without clinical significance at the end of weeks 2 and 6).

**Laboratory testing**

**Blood routine test**: only one patient in the 40 mg/day group had decreases in both leukopenia and neutropenia to a moderate degree, and no treatment was indicated.

**Blood biochemistry testing**

Placebo group: triglyceride increased in one case.

40 mg/day group: four patients had 10 abnormalities: one patient had increase in glutamic-pyruvic transaminase; one patient had increase in total bilirubin and direct bilirubin; one patient had increases in uric acid, creatinine, and triglyceride; one patient had increase in glutamic pyruvic transaminase, aspartate aminotransferase, alkaline phosphatase, and glutamyl transpeptidase.

80 mg/day group: three patients had five abnormalities: one patient had decrease in serum sodium; one patient had increases in total bilirubin and direct bilirubin; one patient had increases in glutamic oxaloacetic transaminase and creatine kinase.

120 mg/day group: two patients had five abnormalities: 1 patient had dyslipidemia, which showed increases in total cholesterol, low-density lipoprotein cholesterol, and triglyceride; one patient had increases in glutamic pyruvic transaminase and glutamyltranspeptidase.

160 mg/day group: two patients had two abnormalities: one patient had increase in triglyceride; one patient had increase in total bilirubin.

**Serum testosterone and prolactin**

There was one case of increase in serum testosterone (40 mg/day group) and three cases of increase in prolactin (one case in 80 mg/day group, one case in 120 mg/day group, and one case in 160 mg/day group). All of them were mild, and no treatment was indicated.

**Urine routine test**

There was no case of a normal routine urine test at baseline that became abnormal after 6 weeks of treatment.
