# Supplementary material for: Efficacy, Safety, and Tolerability of Ansofaxine (LY03005) Extended-Release Tablet for Major Depressive Disorder: A Randomized, Double-Blind, Placebo-Controlled, Dose-Finding, Phase 2 Clinical Trial
Source: Int J Neuropsychopharmacol. 2021 Nov 8;25(3):252–60. doi: 10.1093/ijnp/pyab074 (PMC8929756; doi:10.1093/ijnp/pyab074)
Supplement: pyab074_suppl_Supplementary_Data_S2 [file pyab074_suppl_supplementary_data_s2.docx]

|  | | |
| --- | --- | --- |
|  | | |
| **Ansofaxine Hydrochloride Extended Release Tablet phase 2 clinical trial protocol**  **-A Multicenter, Randomized, Double-Blind, Placebo-controlled, Dose-finding Clinical Trial to Preliminarily Evaluate the Efficacy and Safety of LY03005 Extended-release Tablets for the Treatment of Major Depressive Disorder (MDD)** | | |
|  | **Clinical trial approval number:** | 2015L01158/2015L01159/2015L01160/  2015L01161 |
|  | **Protocol Number:** | LY03005/CT-CHN-204 |
|  | **Leading unit:** | Peking University Sixth Hospital |
|  | **Principal investigator:** | Hongyan ZHANG |
|  | **Sponsor:** | Shandong Luye Pharmaceutical Co., Ltd. |
|  | **Version Number:** | 2.0 |
|  | **Date of version:** | July 1, 2016 |

| **Statement of Confidentiality**  This document contains confidential information of Shandong Luye Pharma Co., Ltd. And you agree to keep this information confidential when accepting or reviewing this document.You may not copy this document or disclose it to others(Except for those as required by applicable laws or regulations or with written permission) and it may not be used for other purpose that are not approved. |
| --- |

**Contact Information**

**Sponsor contact information**

Shandong Luye Pharmaceutical Co., Ltd.

Name: Dr. Guo Shuren, Vice President, Clinical Medical Research Center

Telephone: 010-5281-9352

Mobile: 13501029003

Fax: 010-52819299

Email: guoshuren@luye.cn

Address: No.9 Baoyuan Road, Laishan District, Yantai, Shandong

If you can not reach the contact above, you can contact the one below for all requiremented information related to the study:

Name: Yan ZHANG, Project manager, Clinical Medical Research Center

Mobile: 13910310185

**Researcher contact**

Clinical Research Team: Peking University Sixth Hospital

Name: Hongyan ZHANG, Professor

Office phone number: 010-82013183

Mobile: 13601237138

Fax: 010-82013183

Email: [sally_zhy@sina.com](mailto:sally_zhy@sina.com)

Address: No. 51 North Huayuan Road, Haidian District, Beijing

**Statisticians contact**

Statistical analysis teaching and research section, the 4th Military Medical University of PLA

Name: Jielai XIA, Professor

Mobile: 13571999716

Email: 13571999716@163.com

Address: No. 169 West Changle Road, Xincheng District, Xi’an, Shaanxi

**Modifications to clinical study protocol (version 2.0)**

The main modifications in version 2.0 compared with version 1.1 are as below:

1. As the reporting time of partial laboratory examinations, such as serological examination, is long and unable to be completed within the specified timeframe in the original protocol (version 1.1), the screening/wash-out period is prolonged to Day -14.
2. Course of therapy in the protocol summary: modified from “a total of 7 weeks, including wash-out period of one week and treatment period of 6 weeks” to “a total of 8 weeks, including wash-out period of 2 weeks and treatment period of 6 weeks”.
3. 4.1 Study method: modified from “this study is comprised of two periods: screening and wash-out period (1 week), double-treatment period (6 weeks). Screening eligible subjects with major depressive disorder will enter the one-week placebo wash-out period and receive placebo two tablets, once per day, for consecutive 7 days.” to “this study is comprised of two periods: screening and wash-out period (2 week), double-treatment period (6 weeks). The 1st period is one screening and wash-out period of 8 days (recommended) to up to 14 days, screening eligible subjects with major depressive disorder will enter the one-week placebo wash-out period and receive placebo two tablets, once per day, for consecutive 7 days. ”.
4. Visit 1 in the study flow chart: time modified from “-8~-1d” to “-14~-1d”.
5. Addition of footnote 1 in the flow chart: “screening/wash-out period: Day -14~-1, all the subjects will be given the drugs for wash-out period on Day -8 and enter one 7-day wash-out period (-7 ± 1d)”.
6. Follow-up procedure: 7.1 Screening/wash-out period modified from “Visit 1—— Day -8~-1” to “Visit 1—— Day -14~-1”.
7. The description of secondary efficacy variable “change in visual analogue scale- pain intensity (VAS-PI)” is specified and modified to “change in visual analogue scale- pain intensity (VAS-PI), including overall pain, headache, back pain, extremity or joint pain, abdominal pain and other pain”.
8. The time of AE record in “9. Adverse event reporting” is clarified and mofidied from “in this study, record of adverse event is mainly the subjective symptoms and physicochemical examination after administration” to “in this study, adverse event (serious adverse event and non-serious adverse event) from the signature of informed consent form to the last visit will be recorded”.
9. A small number of editorial changes.

**TABLE OF CONTENTS**

[List of Abbreviations 5](#_Toc32719)

[Abstract 7](#_Toc11811)

[1. Background information 10](#_Toc14642)

[2. Objectives 15](#_Toc2834)

[3. Reference for Protocol Design 16](#_Toc23739)

[4. Trial design 16](#_Toc28823)

[4.1 Test method 16](#_Toc15979)

[4.2 Rationale for Dose Selection 17](#_Toc3305)

[4.3 Rationale for selection of administration method 17](#_Toc15601)

[4.4 Selection basis of control drug 18](#_Toc20483)

[4.5 Set-up of course of therapy 18](#_Toc3133)

[4.6 Sample size calculation 18](#_Toc6769)

[4.7 Randomization method 18](#_Toc32156)

[4.8 Requirement and method for blinding 18](#_Toc26484)

[5. Selection of Subjects 19](#_Toc31183)

[5.1 Inclusion criteria 19](#_Toc30023)

[5.2 Exclusion criteria 19](#_Toc17591)

[5.3 Elimination criteria 21](#_Toc17548)

[5.4 Withdrawal criteria: 21](#_Toc8080)

[5.5 Early Study Termination 22](#_Toc20524)

[6. Dose regimen 22](#_Toc17872)

[6.1 Name and source of investigational product 22](#_Toc10544)

[6.2 Pharmaceutical Presentation 22](#_Toc25118)

[6.3 Drug blinding 23](#_Toc15960)

[6.4 Grouping and administration method 23](#_Toc17419)

[6.5 Dose adjustment 23](#_Toc5504)

[6.6 Drug Accountability 24](#_Toc9319)

[6.7 Drug storage 24](#_Toc11536)

[6.8 Evaluation of compliance with medication 24](#_Toc30057)

[6.9 Concomitant medicationand therapy 24](#_Toc11788)

[7. Follow-up procedure 25](#_Toc25870)

[7.1 Screening/wash-out period 27](#_Toc7035)

[7.2 Baseline 27](#_Toc11082)

[7.3 Double-blind treatment period 28](#_Toc21060)

[8. Evaluation variable(s) 30](#_Toc19216)

[8.1 Efficacy variables 30](#_Toc1202)

[8.2 Safety Indicators 30](#_Toc25649)

[9. Adverse Events Reporting 33](#_Toc30513)

[9.1 AE record(s) 34](#_Toc26023)

[9.2 Criteria for severity assessment of adverse events 34](#_Toc12310)

[9.3 Assessment criteria for the correlation between AEs and investigational product 34](#_Toc10952)

[9.4 Treatment and follow-up of adverse event 35](#_Toc6755)

[9.5 Serious Adverse Event 35](#_Toc9070)

[9.6 Pregnancy 36](#_Toc23526)

[10. Unblinding 37](#_Toc26148)

[11. Data managementand statistical analysis 37](#_Toc24875)

[11.1 Data management 37](#_Toc17606)

[11.2 Statistical analyses 38](#_Toc30225)

[12. Quality control and assurance 41](#_Toc32585)

[13. Ethical requirements 42](#_Toc14346)

[13.1 Good Clinical Practice 42](#_Toc19833)

[13.2 Informed consent 42](#_Toc22344)

[13.3 Early termination of the trial 42](#_Toc32753)

[14. Summary Report 42](#_Toc1640)

[15. Amendment to study protocol 42](#_Toc14750)

[16. Publication 42](#_Toc13400)

[17. Archival of materials 43](#_Toc3721)

[18. References 43](#_Toc19081)

[Attachments 44](#_Toc31165)

[Appendix 1: Hamilton Depression Scale -17 (HAM-D_17_) 44](#_Toc14775)

[Appendix 2: Clinical global impression scale (CGI) 47](#_Toc27517)

[Appendix 3: Hamilton Anxiety Scale (HAMA) 48](#_Toc12734)

[Appendix 4: Visual Analog Scale – Pain intensity (VAS-PI) 49](#_Toc31283)

[Appendix 5: Description of amendment clinical trial protocol 51](#_Toc1372)

List of Abbreviations

| **English Abbreviation** | **Definition** |
| --- | --- |
| 5-HT | 5-hydroxytryptamine |
| AE | Adverse event |
| ALT | Alanine aminotransferase |
| ANCOVA | ANCOVA analysis |
| AST | Glutamic-oxaloacetic transferase |
| AUC | Area under the concentration-time curve |
| BP | Blood pressure |
| CFDA | National Medical Products Administration |
| CGI-I | Clinical global impression scale-Overall improvement |
| CGI-S | Clinical Global Impression scale-Severity |
| C_max_ or Cmax | Maximum plasma concentration |
| Cr | Creatinine |
| CRF | Case Report Form |
| CYP 450 | cytochrome P450 enzyme |
| DA | Dopamine |
| DAT | Dopamine transporter |
| DBP | DBP |
| DSM-IV-TR | Diagnostic and Statistical Manual of Mental Disorders, 4th edition, revised |
| ECG | Electrocardiogram |
| ECT | Electroconvulsive therapy |
| FAS | Full analysis set subset |
| FT3 | Tri-iodothyronine free |
| FT4 | Thyroxine free |
| GCP | Good Clinical Practice |
| h | Hour |
| HAMA | Hamilton Anxiety Scale |
| HAM-D_17_ | Hamilton Depression Scale - 17 |
| LOCF | Last observation carried forward |
| MAOIs | Monoamine oxidase inhibitor |
| MDD | Depressive disorder |
| NaSSA | Noradrenergic and specific 5-serotonergic antidepressant |
| NDRIs | Norepinephrine and dopamine reuptake inhibitor |
| NE | Norepinephrine |
| NET | Norepinephrine transporter |
| NOAEL | No observed-adverse-effect level |
| ODV | *O*-desmethylvenlafaxine |
| PI | PI |
| PPS | Per Protocol Population |
| SAE | Serious Adverse Event |
| SBP | SBP |
| SERT | 5-hydroxytryptamine transporter |
| SNRIs | 5-hydroxytryptamine and norepinephrine reuptake inhibitor |
| SSRIs | Selective Serotonin Reuptake Inhibitor |
| t_1/2_ | Half-life |
| TCAs | Tricyclic antidepressant |
| T_max_ | Time to maximum observed plasma concentration |
| TSH | Thyroid stimulating hormone |

Abstract

| **Sponsor** | Shandong Luye Pharma Co Ltd |
| --- | --- |
| **Investigational product** | Ansofaxine Hydrochloride Sustained-release Tablets |
| **Trial title:** | A Multicenter, Randomized, Double-Blind, Placebo-controlled, Dose-finding Clinical Trial to Preliminarily Evaluate the Efficacy and Safety of Ansufaxine Extended-release Tablets for the Treatment of Major Depressive Disorder (MDD) |
| **Objectives** | Primary objective:  To find the optimal dose of Ansufaxine extended-release tablets in the treatment of MDD.  Secondary objective:  To evaluate the preliminary efficacy and safety of Ansufaxine extended-release tablets for the treatment of MDD, thereby providing a basis for the determination of study design and dosing regimen for a phase 2I clinical trial. |
| **Subject Population** | Patients aged 18~65 years who meet DSM-IV-TR diagnostic criteria for major depressive disorder |
| **Number of subjects** | 5 groups, 52 subjects/group, a total of 260 subjects |
| **Study Methods** | Multicenter, randomized, double-blind, placebo parallel controlled, dose exploratory |
| **Investigational product** | 1. Study drug: Ansofaxine Hydrochloride Extended Release Tablet, specification 40mg, researched, developed and provided by Shandong Luye Pharmaceutical Co., Ltd. 2. Control drug: Simulator for Ansofaxine Hydrochloride Extended-release Tablet (placebo), researched, developed and provided by Shandong Luye Pharmaceutical Co., Ltd. |
| **Group and**  **Dosage and administration** | 1. Trial Grouping:   260 patients with major depressive disorder who meet the enrollment criteria will be randomized to 4 study drug dose groups of and 1 placebo group, i.e., 52 patients each group.   1. Dosage and administration:   Wash-out period: placebo two tablets, once per day, administered under fasted state or after meal at a relatively fixed time in the morning, for consecutive one week.  Treatment period: Ansofaxine Hydrochloride Extended Release Tablet 40mg, 80mg, 120mg, 160mg or placebo orally, once per day, administered under fasted state or after meal at a relatively fixed time in the morning, for consecutive 6 weeks. |
| **Course of therapy** | A total of 8 weeks, including a screening/wash-out period of two weeks and treatment period of 6 weeks. |
| **Visit frequency** | Follow-up at screening, baseline (Day 0), 1, 2, 4 and 6 weeks after administration. |
| **Inclusion criteria** | 1. Male and female outpatients or inpatients aged 18~65 years; 2. Subjects who meet the Diagnostic and Statistical Manual of Mental Disorders, Fourth Edition (DSM-IV-TR) criteria for MDD with a single or recurrent episode(296.2/296.3), without psychotic features; 3. Subjects who have 17-item Hamilton Depression Scale (HAM-D_17_) total score≥20 points at the screening and baseline visit; 4. Subjects who have Depressed Mood of Hamilton Depression Scale (HAM-D_17_) ≥2 points at the screening and baseline visit; 5. Subjects who have clinical Global Impression-Severity Scale (CGI-S) score ≥4 points at the screening and baseline visit; 6. Women of childbearing age (e.g., women who have not experienced sterilization or have had menopause for less than 1 year) who have negative result shown by the urine pregnancy test at the screening and baseline visit; male and female subjects of childbearing age who agree to use a medically acceptable form of contraception for the duration of the study and until at least 28 days after the last oral dose of the study medication; 7. Subjects who voluntarily participate in the trial and sign informed consent forms and who are able to comply with the procedures for scheduled visits, treatments, laboratory tests and other research procedures. |
| **Exclusion criteria** | 1. Subjects who are allergic or known to be allergic to venlafaxine and desvenlafaxine 2. Subjects who have previously failed treatment with venlafaxine at an adequate dose for an adequate time, or patients who have refractory depression and have previously failed treatment with at least 2 types of antidepressants at an adequate dose for an adequate time; 3. Subjects who have greater than 25% change in HAM-D17 total score at baseline from screening visit; 4. Subjects who have clear suicide attempt or behavior with Suicide score in HAM-D17 ≥ 3 points; 5. Pregnant or breastfeeding women, or men and women of childbearing age who do not use a medically acceptable form of contraception or fail to continue contraception until 28 days after the last dose of the study medication; 6. Subjects with DSM-IV-TR axis I diagnoses other than MDD, who receive treatment for such diagnoses (including current or previous diagnoses of anorexia nervosa or bulimia nervosa), or who have been diagnosed with depressive neurosis within the past 2 years;Depressive episode secondary to other psychiatric disorder or somatic disease; 7. Subjects with MDD secondary to other organic or mental disorders; 8. Subjects with a history of seizures (except spasms caused by infantile hyperpyretic convulsions); 9. Subjects who have received electroconvulsive therapy (ECT) within 3 months prior to screening or need current ECT treatment per investigator’s judgment, have received systematic psychotherapy (interpersonal relationship therapy, dynamic therapy, cognitive behavioral therapy) within 3 months of screening, have received transcranial magnetic stimulation (TMS) 3 months prior to screening, or have received light therapy 2 weeks prior to screening; 10. Subjects who have regularly taken anti-depressants within 2 weeks prior to screening, or have withdrawn from psychotropic drugs for less than 7 half-lives (at least 2 weeks for monoamine oxidase inhibitors and at least 1 month for fluoxetine) before randomization/enrollment; 11. Subjects with a history of serious unstable cardiovascular, hepatic, renal, blood or endocrine diseases; 12. Subjects with hypertension whose blood pressure is poorly controlled (SBP≥140 mmHg or DBP≥90 mmHg at the screening and baseline visit); 13. Subjects with a history of gastrointestinal diseases known to interfere with drug absorption or excretion, or those with a history of surgery that is known to interfere with drug absorption or excretion; 14. Subjects with a history of increased intraocular pressure or narrow angle glaucoma; 15. Subjects with clinically significant abnormal results as considered by the investigator shown by a physical examination, laboratory test or urine drug test (e.g., ALT or AST> 1.5 times the upper limit of normal; Creatinine clearance > the upper limit of normal; abnormal measurements of thyroid function which are of clinical significance); 16. Subjects with Electrocardiogram (ECG) abnormalities that are clinically significant at screening and baseline, and investigators believe that it is inappropriate for the subjects to be enrolled, such as QTc interval >450 ms for male and QTc interval >460 ms for female; 17. Subjects who have participated in clinical trials on other drugs within 3 months prior to screening; 18. Subjects with serious acute or chronic diseases, mental illnesses or clinically significant abnormalities as shown in laboratory tests, of which investigators believe that the subjects are not suitable for this study |
| **Efficacy evaluation**  **Variables** | Primary efficacy variable:  Changes from baseline in the total score of 17 items of Hamilton Depression Scale (HAM-D_17_) at the end of treatment  Secondary efficacy variables:   - Change in the Clinical Global Impression Scale-Improvement (CGI-I) score at the end of treatment - Change from baseline in the Clinical Global Impression Scale-Severity (CGI-S) score at the end of treatment - Change from baseline in the Hamilton Anxiety Scale (HAMA) at the end of treatment - Change from baseline in the Visual Analog Scale of Pain Intensity (VAS-PI) score at the end of treatment - Change from baseline in HAM-D_17_ factor scores at the end of treatment - HAM-D_17_ response rate (≥ 50% reduction from baseline in HAM-D17 total score) - HAM-D_17_ remission rate ( HAM-D17 total score ≤7) |
| **Safety Evaluation Variables** | - Adverse events - Vital signs, physical examination, laboratory tests (hematology, urinalysis, blood chemistry test and serological test), 12-lead ECG findings |
| **Statistical analyses** | To evaluate the LY03005 extended-release tablets effect compared to placebo on change from baseline in the HAM-D17 total score at end of week 6 by using analysis of covariance (ANCOVA) model, statistical tests were conducted using two-sided test and the difference was considered statistically significant (unless otherwise specified) when P value was ≤ 0.10. Adjustment was not made when using multiple tests. Intergroup differences on change from baseline in the HAM-D17 total score were compared using ANCOVA, including the HAM-D17 total score at baseline as a covariate, and adjusted by site effect. Difference of LSMEANs compared to placebo group and associated 90% Tukey-Kramer-adjusted confidence intervals will be tabulated.  The type and frequency of adverse events and relationship with the investigational drug would be summarized. Subjects discontinued the study due to AEs and those with severe or serious AEs were noted.  The shift tables will be used for the laboratory tests, ECG findings, physical examination (clinical significance will be based on the investigator’s judgment). Abnormal findings of clinical significance were listed.  Changes in the vital signs from baseline at each visit were described and descriptive statistics were calculated, which include the number of subjects, mean, standard deviation, median, minimum and maximum. Paired t-test was used to analysis the difference between post-treatment and baseline in the same treatment group. The differences of change from baseline between treatments groups were analyzed by using ANCOVA or rank sum test. |

**Ansofaxine Hydrochloride Extended Release Tablet phase 2 clinical trial protocol**

**-A Multicenter, Randomized, Double-Blind, Placebo-controlled, Dose-finding Clinical Trial to Preliminarily Evaluate the Efficacy and Safety of LY03005 Extended-release Tablets for the Treatment of Major Depressive Disorder (MDD)**

# Background information

Major depressive disorder (MDD) is a common and serious mental illness with high incidence, high recurrence and high suicide rate. It is characterized by low mood, lack of interest and pleasure, self-blame or inferiority, sleep disorders, loss of appetite, low energy loss and inattention and may be chronic and recurrent, which can lead to loss of work ability and even suicide.

Currently commonly used antidepressants include tricyclics (TCAs), monoamine oxidase inhibitors (MAOIs), selective serotonin reuptake inhibitors (SSRIs), serotonin and norepinephrine reuptake inhibitors (SNRIs), norepinephrine-dopamine reuptake inhibitors (NDRIs) and noradrenergic and specific serotoninergic antidepressants (NaSSA). As a norepinephrine (NE) and 5-hydroxytryptamine (5-HT) dual uptake inhibitor, venlafaxine hydrochloride (trade name Effexor®, marketed by Wyeth in 1993) and desvenlafaxine (trade name PRISTIQ®, marketed by Wyeth in 2008) are widely used in clinical practice but it has shortcomings such as slow onset of action, sexual dysfunction and lack of improvement in pleasure.

The proposed LY03005 extended-release tablet (Ansofaxine hydrochloride, LPM570065) is a new class of antidepressant developed by Shandong Luye Pharmaceutical Co., Ltd., which is para-methyl benzoate of the O-desvenlafaxine (ODV) and has less hydrophilic groups than ODV and higher fat solubility and membrane permeability. After entering the body, Ansofaxine hydrochloride can enter the brain with its metabolite ODV and the two can play a therapeutic role together in the selective hypothalamus area of the target organ. Compared with PRISTIQ®, LY03005 extended-release tablets have not only improved bioavailability but also increased inhibition on dopamine (DA) uptake. It is a potential three-uptake inhibitor that will have a rapid onset that improves the loss of pleasure and sexual dysfunction, cognitive function and reward-driven and goal-oriented behaviors.

**Pharmacological action**

Ansofaxine hydrochloride extended-release tablets release the drug into the blood stream continuously. When the drug is released, both ansofaxine and its metabolite ODV enter the brain, distribute selectively in the target organ hypothalamus with comparable concentrations, and work together to achieve therapeutic effects. Ansofaxine hydrochloride displayed high affinities for the dopamine transporter (DAT), the norepinephrine transporter and the serotonin transporter (SERT). It had significant inhibitory effects on the reuptake of DA, NE and 5-HT. After administered to rats, ansofaxine hydrochloride revealed an overall effect that is characteristic of a tri-inhibitor on the reuptake of DA, NE and 5-HT. Obvious occupancies of DAT, NET and SERT by ansofaxine hydrochloride in the rat brain were also seen. A single intragastric administration of ansofaxine hydrochloride was able to: 1) significantly increase the head twitches induced by 5-hydroxytryptophan (5-HTP) at sub-threshold doses in rats; 2) suppress the decrease in body temperature induced by apomorphine hydrochloride in mice; 3) increase the toxicity of yohimbine at sublethal doses in mice; 4) reduce the immobility time in the forced swimming test performed in rats and in the tail suspension test performed in mice; 5) raise the time spent by anxious rats in the open arms of an elevated zero maze; 6) inhibit the writhing response in mice induced by acetic acid;7)attenuate the central and peripheral pains in the formalin test conducted in mice. Continuous intragastric administration of ansofaxine hydrochloride was found to markedly inhibit the elevated spontaneous locomotor activities in rats following olfactory bulb removal;increase the consumption of sugared water; reduce circulating corticosterone levels; and increase circulating testosterone levels.

**[Toxicological studies]**

Ansofaxine hydrochloride inhibited the potassium channels of Chinese hamster ovary (CHO) cells stably expressing the hERG gene in vitro, with an IC50 of 2.43 μM. However, no change in the corrected QT interval (QTc) on the electrocardiography(ECG) was observed in either the safety pharmacology study or the subchronic toxicity study. Meanwhile, ODV, but not ansofaxine, was detected in the plasma of the Cynomolgus monkeys treated with ansofaxine hydrochloride extended-release tablets in the pharmacokinetic study, suggesting that ansofaxine hydrochloride has no cardiotoxicity.

Ansofaxine hydrochloride had no irritating effect on the gastrointestinal tract. A single dose of ansofaxine hydrochloride administered intragastrically to rats did not have obvious effects on the systemic functions of the body. No noticeable impact on the locomotor activities or the motor coordination was found in mice. No synergistic action with pentobarbital sodium at subthreshold doses was identified. No marked effects were observed on the blood pressure, heart rate, ECG, or breathing in the Cynomolgus monkeys under anesthesia.

In the acute toxicity study conducted in Sprague Dawley (SD) rats, a single, very high dose of ansofaxine hydrochloride resulted in the death of some animals. Some rats exhibited symptoms/signs that may be linked to the drug’s mechanism of action, including tremor/shiver, convulsion, spasm, redness of the extremities and the ears, decreased spontaneous movements, abnormal posture, lethargy, and prostrate posture. No obvious abnormality was noted for the rats treated with ansofaxine hydrochloride at 500 mg/kg (31.3 times the maximum recommended therapeutic dose, MRTD). No abnormality was observed in the Cynomolgus monkeys intragastrically administered a single dose of ansofaxine hydrochloride at 500 mg/kg (62.5 times the MRTD).

In one of the subchronic toxicity study of rats, the animals were treated with an intragastric administration of ansofaxine hydrochloride for 28 consecutive days. At the very high dose, a few animals experienced deaths and damages to the respiratory tract. One death was observed in rats receiving a dose of 300 mg/kg (18.8 times the MRTD). These deaths were unrelated to systemic toxicities of the drug. Instead, they may be attributable to the cumulative damages resulted from the repeated inhalation of ansofaxine hydrochloride into the lungs when the drug was administered. In the animals treated at a dose of 300 mg/kg (18.8 times the MRTD) or greater, mild lesions on the eyelids, damages on the lens and vitreous opacities were observed. These damages may be associated with the activation of the 5-HT receptors resulted from the drug’s inhibitory effect on 5-HT reuptake. No obvious abnormalities were identified in the animals receiving a dose of 100 mg/kg (6.3 times the MRTD). The toxicokinetic study conducted at the same time showed no obvious drug accumulation in any of the dosage groups with continuous dosing for 28 days. The no-observed-adverse-effect level (NOAEL) was 100 mg/kg, which was converted based on body surface area and equivalent to 6.3 times the MRTD.

In the subchronic toxicity study in Cynomolgus monkeys, ansofaxine hydrochloride was orally administered for 28 days continuously. Sporadic (2 out of 8), reversible thymic atrophy was observed in the animals in the 200 mg/kg dosage group (25 times the MRTD). This damage may be related to the excessive inhibition on the reuptake of NE and 5-HT caused by an overly high dose of the drug, which can lead to decreased appetite and weight loss. These appetite and weight changes can then stimulate the adrenal cortex and trigger the stress response. No obvious abnormalities were identified in the animals receiving a dose of 60 mg/kg (7.5 times the MRTD). The toxicokinetic study in these Cynomolgus monkeys showed no gender difference and no obvious drug accumulation in any of the dosage groups with continuous dosing for 28 days. The NOAEL was 60 mg/kg, which was converted based on body surface area and equivalent to 7.5 times the MRTD.

In the other subchronic toxicity study in rats, ansofaxine hydrochloride was intragastrically administered for 91 days continuously at three different dosages (30, 100, 300 mg/kg). One animal in the 300 mg/kg (18.8 times the MRTD) dosage group experienced near death that was spontaneous and unrelated to the treatment. Other than a one-time, transient ptyalism in all dosage groups and a transient, slight weight loss in the male animals in the 300 mg/kg group, no obvious abnormalities was noted in any of the following examinations or tests: food consumption, ophthalmology, hematology,blood biochemical analysis, hormone levels, urinalysis, organ weights and ratios, gross anatomy, and histopathology. The toxicokinetic study in rats showed no obvious drug accumulation in any of the dosage groups with continuous dosing for 91 days. The NOAEL was 300 mg/kg, which was converted based on body surface area and equivalent to 18.8 times the MRTD.

In the subchronic toxicity study in Beagle dogs, ansofaxine hydrochloride extended-release tablets were orally administered for 91 days continuously at three different dosages (50, 100, 200 mg/kg). Except for transient weight loss and reduced food consumption in the female dogs in the 200 mg/kg group, no obvious abnormalities was noted in the general health of the animals or any of the following examinations or tests: ophthalmology, hematology, blood biochemical analysis, hormone levels, urinalysis, bone marrow smear, organ weights and ratios, gross anatomy, and histopathology. The toxicokinetic study in these dogs showed no obvious drug accumulation in any of the dosage groups with continuous dosing for 91 days. The NOAEL was 200 mg/kg，which was converted based on body surface area and equivalent to 37.5 times the MRTD.

The genetic toxicology study indicated that ansofaxine hydrochloride was not mutagenic and did not induce chromosomal aberration in cells, nor did it cause DNA damages or chromosomal aberration in mice.

The reproductive toxicity studies in rats did not find any toxicologically relevant abnormalities or drug-related toxicopathological changes under gross or microscopic examinations, except for reduced embryo implantations in the 300 mg/kg (18.8 times the MRTD) group. The NOAEL was 100 mg/kg, which was converted based on body surface area and equivalent to 6.3 times the MRTD.

The reproductive toxicity study of embryo fetal development (EFD) in rabbits found no obvious changes in pregnant animals in any dosage group (15.6,52.0,or 156.0mg/kg);nor did the drug have any obvious effects on the reproductive functions of the pregnant rabbits,embryo formation, or or the development of embryo and fetus. The toxicokinetic study in these rabbits showed no obvious drug accumulation in the body. The NOAEL for the pregnant rabbits and for the development of embryo and fetus was 156 mg/kg, which was converted based on body surface area and equivalent to 18.8 times the MRTD.

**Non-clinical pharmacokinetics**

After entering the body, ansofaxine hydrochloride was rapidly metabolized into ODV. Ansofaxine was nearly undetectable in the blood. After a single dose of ansofaxine hydrochloride (4 - 16 mg/kg in the rats; 10 - 30 mg/kg in the Cynomolgus monkeys) was administered intragastrically, the Cmax and AUC of ODV were both positively correlated with the dosages. The rate of drug clearance was similar to what was observed when equal molar doses of ODV were administered intragastrically. No gender differences were seen in the rats or the monkeys.

Compared with PRISTIQ^®^, the relative bioavailability of ansofaxine hydrochloride extended-release tablets was 131% (calculated based on the measured levels of the metabolite ODV). When equal molar doses of ansofaxine hydrochloride extended-release tablets and solution were administered to the Cynomolgus monkeys orally, the tablet group showed significantly greater t1/2 and T_max_ than the solution group, which confirms the slow-release character of the extended-release tablets. The accumulation factor after multiple dosing of ansofaxine hydrochloride extended-release tablets was 1.15, indicating no obvious accumulation of ansofaxine hydrochloride in the body.

After ansofaxine hydrochloride was administered to the rats, both ansofaxine and its metabolite ODV were detectable in tissues. Ansofaxine and ODV were rapidly distributed to a wide range of tissues, but particularly selectively to the target organ inside the brain, hypothalamus (C_hypothalamus_ >> C_brain_). At 0.25 h and 1 h, the concentrations of ansofaxine and ODV in the hypothalamus were comparable. Both ansofaxine and ODV were mostly cleared from all tissues 12 hours after dosing (with concentrations less than 10% of the C_max_), suggesting no obvious accumulation.

The study of ansofaxine hydrochloride metabolism indicated that it is first metabolized into ODV, and then is further metabolized into the same ODV metabolite as previously reported in the literature. No other metabolites were identified.

Ansofaxine hydrochloride showed no obvious inducing effect on hepatic microsomal enzymes CYP1A2, CYP2D6, CYP2C9, CYP3A4 and CYP2C19 in rats. It had no inhibitory effect on human recombinant CYP1A2, CYP 2C9, CYP 2C19, CYP 2D6 and CYP 3A4.

The urinary excretion of ODV reached a plateau 24 hours after ansofaxine hydrochloride was administered to rats (greater than 90% of the total excreted amount in 144 hours). The main excretion routes were urine and feces. About 19.5% (± 6.8%) of ODV was excreted in the free form and 75.6% (± 12.6%) was excreted in the conjugated form. The total excretion of metabolites in urine and feces was 95.1% (± 19.6%).

**CLINICAL PHARMACOLOGY**

1. Evaluation of the tolerance and pharmacokinetics of a single oral dose of ansofaxine hydrochloride extended-release tablet in healthy volunteers in a randomized, double-blind, placebo-controlled, dose-escalation study.

Seventy-two male and female healthy volunteers were enrolled in this trial, including 60 subjects receiving Ansofaxine Hydrochloride Extended release Tablet (study drug) orally and 12 subjects receiving placebo. 20 case-times of adverse events occurred in 14 subjects receiving study drug, and 9 case-times of adverse events were possibly related to the study drug, including 4 case-times of nausea, 1 case-time each of dizziness, weakness, diarrhea, supraventricular arrhythmia (unconfirmed) and sinus tachycardia. All the adverse events possibly related to the study drug were mild and spontaneously relieved without treatment. No clinically significant change related with the study drug was seen in physical examination, blood oxygen saturation and laboratory examination. The study results showed a good safety and tolerability for orally single dose of Ansofaxine Hydrochloride Extended release Tablet in the dose range of 20~200 mg.

At the orally single dose of 20, 40, 80, 120, 160 and 200 mg Ansofaxine Hydrochloride, Ansofaxine was rapidly metabolized to the active metabolite *O-*demetylvenlafaxin, a very low concentration of parent drug could be detected in individual plasma and urine samples in 80~200 mg dose group, the median T_max_ was 6.00~8.00 hours, t_1/2_was 8.30~11.29 hours, C_max_ and AUC were increased approximately in proportion to dose increase, as calculated by *O-*demetylvenlafaxin, and about 50% oral drug was excreted via kidney in a form of *O-*demetylvenlafaxin.

1. Evaluation of 2-period, 2 sequence an crossover,comparative pharmacokinetics study of ansofaxine hydrochloride extended-release tablet after fasting and after fed in healthy volunteers

Twelve healthy volunteers were enrolled in this trial, including half men and half women. A total of 5 case-times of adverse events occurred in 3 subjects, only one was possibly related with the study drug and appeared nausea, mild in intensity and spontaneously relieved without treatment. The study results showed a good safety and tolerability for orally single dose of Ansofaxine Hydrochloride Extended release Table 120mg under fasted state and after high-fat meal.

Compared with the administration under fasted state, the T_max_was prolonged, C_max_was elevated and relative bioavailability was about 106% for *O-*demetylvenlafaxin in healthy subjects, after oral administration of Ansofaxine Hydrochloride Extended release Table 120 mg following meals.

1. Evaluation of the tolerance and pharmacokinetics of single dose vs. multiple doses of ansofaxine hydrochloride extended-release tablet in healthy volunteers in a randomized,double-blind,placebo-controlled,dose-escalation study.

A total of 48 male and female healthy volunteers were enrolled in this trial, including 40 subjects receiving Ansofaxine Hydrochloride Extended release Table orally and 8 subjects receiving placebo. 24 case-times of adverse events occurred in 15 volunteers receiving Ansofaxine Hydrochloride Extended release Table, including a total of 16 case-times of adverse events possibly related with the study drug, the common related adverse events were 5 case-times of nausea, 3 case-times of vomiting and 3 case-times of diarrhea, the other related adverse events also included 2 case-times of dizziness, 2 case-times of elevation of total bilirubin and 1 case-time of elevated alanine aminotransferase, the majority of them were mild in intensity and spontaneously relieved without treatment. No clinically significant change related with the study drug was seen in vital signs, physical examination, 12-lead ECG and blood oxygen saturation. The study results showed a good safety and tolerability for oral Ansofaxine Hydrochloride Extended release Table once per day for consecutive 7 days, in the dose range of 40~160 mg.

Results of the study on Ansofaxine Hydrochloride Extended release Table after orally multiple doses showed the median T_max_ was 3~6 h, mean t_1/2_was 9.09~10.04 h, the systemic exposure was mildly increased in each dose group and the accumulation factor was about 1.20, in the dose range of 40~160 mg/day. The plasma concentration of ODV could reach steady state after consecutive administration for three days. The steady-state C_max_ and AUC of ODV were dose proportional.

This clinical trial was conducted in accordance with the clinical trial approval letter issued by China Food and Drug Administration (CFDA) on June 16, 2015 (approval letter number: 2015L01158/2015L01159/2015L01160/2015L01161).

# Objectives

Primary objective: To find the optimal dose of LY03005 extended-release tablets in the treatment of MDD.

Secondary objective: To evaluate the preliminary efficacy and safety of LY03005 extended-release tablets for the treatment of MDD, thereby providing a basis for the determination of study design and dosing regimen for a phase 2I clinical trial.

# Reference for Protocol Design

1. Technical guideline on the drug clinical trial for treatment of major depressive disorder (exposure draft) (2013)
2. Measures for Drug Registration (2007)
3. Good Clinical Practice (GCP) (2003)
4. Rational for study of the Ansofaxine Hydrochloride Extended-release Tablets
5. The preclinical pharmacokinetic study of Ansofaxine Hydrochloride Extended release Tablets
6. The preclinical pharmacological and toxicological studies of Ansofaxine Hydrochloride Extended release Tablets
7. CSR of a randomized, double-blind, placebo-controlled, dose escalation trial on the tolerability and pharmacokinetics of Ansofaxine Hydrochloride Extended release Tablets in healthy volunteers following single dose orally
8. CSR of a two-cycle, two-sequence, randomized, crossover trial to compare the pharmacokinetics of oral Ansofaxine Hydrochloride Extended release Tablet in healthy volunteers under fasted state and after meals
9. CSR of a randomized, double-blind, placebo-controlled, dose escalation trial on the tolerability and pharmacokinetics of Ansofaxine Hydrochloride Extended release Tablets in healthy volunteers following single dose and multiple doses orally

# Trial design

## Test method

This study was a multicenter, randomized, double-blind, placebo parallel-controlled, dose-finding phase 2 clinical trial to find the optimal dose of LY03005 extended-release tablets for the treatment of MDD and to evaluate the preliminary efficacy and safety, providing a basis for the design of phase 2I clinical trials and the determination of dosing regimens.

This study consisted of two periods: a screening and washout period of up to 2 weeks (8 days recommended) and a double-blind treatment period of 6 weeks. The screening and washout period were expected to last from 8 days (recommended) to 2 weeks. After screening, eligible patients with MDD entered a 1-week placebo washout period, during which they took 2 placebo tablets orally once a day in the morning for 7 consecutive days. 260 patients with MDD who still met the inclusion criteria after the placebo washout then entered a double-blind treatment period of 6 weeks. They were randomly assigned to placebo or one of four fixed does LY03005 extended-release tablets (40, 80, 120 or160mg/day) using a 1:1:1:1:1 randomization schedule. Subjects took 4 tablets of test drug or placebo once a day and returned for follow-up visits at the weekends of study week 1, 2, 4 and 6.

Efficacy endpoints included 17-item Hamilton Depression Scale (HAM-D17), Hamilton Anxiety Rating Scale (HAM-A), Clinical Global Impression (CGI) and Visual Analog Scale for Pain Intensity (VAS-PI). Safety evaluation included adverse events, vital signs, physical examination, laboratory tests (hematology, urinalysis, blood chemistry test and serological test), and 12-lead ECG.

## Rationale for Dose Selection

1. The preclinical pharmacodynamic study showed that Ansofaxine Hydrochloride could significantly improve the depressive state in animals in depression evaluation model, the minimum effective dose was 4 mg/kg in rats and 8 mg/kg in mice, the calculated human equivalent dose was 38.4 mg, thus the minimum dose was determined as 40 mg for the phase 2 dose-exploratory study.
2. The specifications of PRISTIQ^®^ in clinical use are 25 mg, 50 mg and 100 mg per tablet, the clinically recommended dose is 50 mg or above per day, however, it is indicated in the package insert as well that no additional benefit is seen at the dose >50 mg. The dose exploration in this study will refer to the clinical dose of PRISTIQ^®^, 80 mg Ansofaxine Hydrochloride Extended Release Tablet is comparable to 50 mg PRISTIQ^®^ (containing Desvenlafaxine succinate 76 mg, 50 mg as calculated by ODV), 160mg Ansofaxine Hydrochloride Extended Release Tablet is comparable to 100 mg PRISTIQ^®^ (containing Desvenlafaxine succinate 152 mg, 100 mg as calculated by ODV), as calculated by equimolar ODV. The highest dose is set as 160 mg/day.
3. Results of the completed phase I clinical trial on tolerability and pharmacokinetics showed a good safety and tolerability for Ansofaxine Hydrochloride Extended Release Tablet once per day in the dose range of 20~200 mg/day following orally single dose, or once per day in the dose range of 40~160 mg/day following orally multiple doses, for consecutive 7 days.

In summary, the doses to be explored in the phaseII clinical trial on Ansofaxine Hydrochloride Extended Release Tablet are 40, 80, 120 and 160 mg/day.

## Rationale for selection of administration method

The study drug Ansofaxine Hydrochloride Extended Release Tablet is one extended release formulation, the available pharmacokinetic study results showed the median T_max_was 3~6 hours and t_1/2_was 9.09~10.04 hours after orally multiple dose of 40, 80, 120 and 160 mg, thus the dose interval was set as once per day.

Results of the completed study to compare the pharmacokinetics of oral Ansofaxine Hydrochloride Extended Release Tablet in healthy volunteers under fasted state and after high-fat meal showed that the time to peak concentration was delayed, peak concentration was increased and relative bioavailability was 106% for ODV after oral administration of Ansofaxine Hydrochloride Extended Release Tablet 120mg following high-fat meal, indicating small effect of diet (high-fat meal) on the bioavailablity of the active metabolite of Ansofaxine Hydrochloride.

In summary, the administration method of Ansofaxine Hydrochloride Extended Release Tablet is once per day, at a relatively fixed time in the morning, under fasted state of after meal.

## Selection basis of control drug

The primary objective of this study is to explore the preliminary antidepressant effect of LY03005 extended-release tablets, analyze the dose-effect relationship, and determine the clinical effective dose range and the best effective dose. In order to fully evaluate the efficacy and safety of LY03005 extended-release tablets in the treatment of depression, placebo was used as the control.

## Set-up of course of therapy

In accordance with the technical guideline on the drug clinical trial for treatment of major depressive disorder (exposure draft) (2013), 6-8 weeks is recommended as the course of therapy for acute phase of major depressive disorder, thus the course of therapy is set as 6 weeks in this study.

## Sample size calculation

This is one phase 2 dose-exploratory study, according to the requirements of CFDA drug regulatory regulationsat, at least 200 valid cases need to be completed, and considering a (20-30)% fall-off rate, a total of 260 cases are expected to be enrolled in consideration of the possible drop-out rate of 30% during the study, and 1:1:1:1:1 randomized to Ansofaxine Hydrochloride Extended Release Tablet 40, 80, 120 and 160 mg dose groups or placebo group, there will be at least 52 screening eligible cases in each group.

## Randomization method

MagMin minimized dynamic randomization system (<http://rct.fmmukq.com>) is used for dynamic randomization. The randomized algorithm is Pocock & Simon. After verifying that the patient is eligible per inclusion criteria and exclusion criteria, the Investigator will log in the MagMin randomization system using the account and password and get the assignment of unique randomization number and drug number for the patient. Eligible patients will be randomized 1:1:1:1:1 to receive LY03005 40mg/day, 80mg/day, 120mg/day, 160mg/day or placebo, respectively. The baseline HAM-D_17_, gender and age score will be used as important prognostic factors for baseline balancing across groups. Within each randomization, patients will be stratified for:

- HAM-D17 total score at baseline (20-30, 31-40, ≥41)
- Gender (Male, Female)
- Age, years (18-30, 31-40, 41-50, 51-65)

## Requirement and method for blinding

Double-blind, single-dummy technique is used. The drug randomization listsare generated by the independent 3rd party using SAS code. A randomized block schedule will be used for the drug randomization lists. The study drug will be manufactured, packaged and provided by Shandong Luye Pharma Co., Ltd., and then will be coded uniformly by the independent 3rd party per the drug randomization lists. In accordance with the drug number replied from MagMin randomization system, subjects will be given the corresponding drugs. Any drug use method that violates this rule will be regarded as violation. Drug number will remain unchanged throughout the study. Each patient will be provided by investigators with the study drug of the same number in divided doses.

# Selection of Subjects

## Inclusion criteria

1. Male and female outpatients or inpatients aged 18~65 years;
2. Subjects who meet the Diagnostic and Statistical Manual of Mental Disorders, Fourth Edition (DSM-IV-TR) criteria for MDD with a single or recurrent episode(296.2/296.3), without psychotic features;
3. Subjects who have 17-item Hamilton Depression Scale (HAM-D_17_) total score≥20 points at the screening and baseline visit;
4. Subjects who have Depressed Mood of Hamilton Depression Scale (HAM-D_17_) ≥2 points at the screening and baseline visit;
5. Subjects who have clinical Global Impression-Severity Scale (CGI-S) score ≥4 points at the screening and baseline visit;
6. Women of childbearing age (e.g., women who have not experienced sterilization or have had menopause for less than 1 year) who have negative result shown by the urine pregnancy test at the screening and baseline visit; male and female subjects of childbearing age who agree to use a medically acceptable form of contraception for the duration of the study and until at least 28 days after the last oral dose of the study medication;
7. Subjects who voluntarily participate in the trial and sign informed consent forms and who are able to comply with the procedures for scheduled visits, treatments, laboratory tests and other research procedures.

## Exclusion criteria

1. Subjects who are allergic or known to be allergic to venlafaxine and desvenlafaxine
2. Subjects who have previously failed treatment with venlafaxine at an adequate dose for an adequate time, or patients who have refractory depression and have previously failed treatment with at least 2 types of antidepressants at an adequate dose for an adequate time;
3. Subjects who have greater than 25% change in HAM-D17 total score at baseline from screening visit;
4. Subjects who have clear suicide attempt or behavior with Suicide score in HAM-D17 ≥ 3 points;
5. Pregnant or breastfeeding women, or men and women of childbearing age who do not use a medically acceptable form of contraception or fail to continue contraception until 28 days after the last dose of the study medication;
6. Subjects with DSM-IV-TR axis I diagnoses other than MDD, who receive treatment for such diagnoses (including current or previous diagnoses of anorexia nervosa or bulimia nervosa), or who have been diagnosed with depressive neurosis within the past 2 years; Depressive episode secondary to other psychiatric disorder or somatic disease;
7. Subjects with MDD secondary to other organic or mental disorders;
8. Subjects with a history of seizures (except spasms caused by infantile hyperpyretic convulsions);
9. Subjects who have received electroconvulsive therapy (ECT) within 3 months prior to screening or need current ECT treatment per investigator’s judgment, have received systematic psychotherapy (interpersonal relationship therapy, dynamic therapy, cognitive behavioral therapy) within 3 months of screening, have received transcranial magnetic stimulation (TMS) 3 months prior to screening, or have received light therapy 2 weeks prior to screening;
10. Subjects who have regularly taken anti-depressants within 2 weeks prior to screening, or have withdrawn from psychotropic drugs for less than 7 half-lives (at least 2 weeks for monoamine oxidase inhibitors and at least 1 month for fluoxetine) before randomization/enrollment;
11. Subjects with a history of serious unstable cardiovascular, hepatic, renal, blood or endocrine diseases;
12. Subjects with hypertension whose blood pressure is poorly controlled (SBP≥140 mmHg or DBP≥90 mmHg at the screening and baseline visit);
13. Subjects with a history of gastrointestinal diseases known to interfere with drug absorption or excretion, or those with a history of surgery that is known to interfere with drug absorption or excretion;
14. Subjects with a history of increased intraocular pressure or narrow angle glaucoma;
15. Subjects with clinically significant abnormal results as considered by the investigator shown by a physical examination, laboratory test or urine drug test (e.g., ALT or AST> 1.5 times the upper limit of normal; Creatinine clearance > the upper limit of normal; abnormal measurements of thyroid function which are of clinical significance);
16. Subjects with Electrocardiogram (ECG) abnormalities that are clinically significant at screening and baseline, and investigators believe that it is inappropriate for the subjects to be enrolled, such as QTc interval >450 ms for male and QTc interval >460 ms for female;
17. Subjects who have participated in clinical trials on other drugs within 3 months prior to screening;
18. Subjects with serious acute or chronic diseases, mental illnesses or clinically significant abnormalities as shown in laboratory tests, of which investigators believe that the subjects are not suitable for this study

## Elimination criteria

For those subjects enrolled into this clinical study, they were removed from this trial if one of the following criteria is met:

(1) Subjects who did not meet the inclusion criteria or met the exclusion criteria;

(2) Subjects who did not have any medication records;

(3) Subjects with no records of efficacy and safety information after the baseline.

## Withdrawal criteria:

### Involuntary withdrawal

1. Subjects with an allergic reaction or a serious adverse event woul be withdrawn per physicians’ judgment;
2. Subjects who contract other serious diseases during the study;
3. Subjects who were unmasked during the trial;
4. Subjects with poor adherence or severe non-compliance with the study procedure, which will affect the safety and efficacy evaluation;
5. Suicide, conversion of mania or obvious psychotic symptoms during the study;
6. Major protocol violations, affecting the final efficacy and safety evaluation;
7. Subject is pregnant during the study;
8. Subjects who meet any of the following criteria:

QTc>500ms

Change from baseline: QTc>60ms

The above criteria should be based on the average result of three 12-lead ECG QTc. For example, if an ECG shows an extension of the QT interval, two additional ECGs should be obtained in a short timeof period. The average of QTc of the three ECGs should be used to determine if the subject should stop the study treatment.

1. In the circumstances that investigatorsbelieve it is necessary to withdraw the subjects from the trial.

### Voluntary withdrawal

1. The subject who were unwilling to continue the clinical trial and withdraw theinformed consent form;
2. Lost to follow-up;
3. Other conditions in which the subject withdrew from the trial (e.g., changes in placeof residence, making it impossible to continue medication and follow-up).

For the withdrawal with any reasons, the efficacy and safety data should be obtained as much as possible. The reason of withdrawal should be recorded on the original medical records and CRFs.

## Early Study Termination

Termination of the study means that the clinical trial has not been completed according to the plan and it is stopped in the middle.

(1) If serious safety problems are found during the study, clinical trials should besuspended in time.

(2) The investigational drug was found to be less effective or even ineffective duringthe studyand it has no clinical value.

(3) In the trial, it was found that the clinical trial protocol had major errors or seriousdeviations occurred during the implementation and it will be difficult to evaluate thedrug efficacy.

(4) Sponsor or administrative department requests study suspension.

# Dose regimen

## Name and source of investigational product

Study drug: Ansofaxine Hydrochloride Extended Release Tablet, specification: 40mg; batch number 150702, valid until June 2017, provided by Shandong Luye Pharma Co., Ltd., and meeting the quality standard for clinical trials.

Control drug: Simulator for Ansofaxine Hydrochloride Extended-release Tablet (placebo), not containing any drug component, consistent appearance with Ansofaxine Hydrochloride Extended Release Tablet, batch number 150701, valid until June 2017, provided by Shandong Luye Pharma Co., Ltd., and meeting the quality standard for clinical trials.

## Pharmaceutical Presentation

All the drugs for the clinical trial will be packaged as required for the clinical trial. The content on the packing label includes drug name, amount, indication, administration and dosage, storage condition, sponsor and the word “use for clinical trial only”.

The drug in wash-out period is one small box packed separately, loaded with the drug Ansofaxine Hydrochloride Extended Release Tablet analogue for the 7-day wash-out period.

The drug in treatment period is one big package with four separate small packages, one separate small package will be distributed at each visit, each small package contains the study drug packed with aluminum plastic, the time window has been considered for drug loading. The specific administration method and precautions have been noted on the large package and small package drug boxes.

## Drug blinding

This is one multicenter, randomized, double-blind, placebo parallel-controlled clinical study. The random number table is provided by statisticians and generated using SAS software Plan process. The drug blinding will be completed by the personnel unrelated with this clinical study.

## Grouping and administration method

Wash-out period (Day -7 ~-1): placebo two tablets once per day, at a relatively fixed time in the morning, under fasted state or after meal.

Treatment period (Day 1 ~ 42): see Table 1 for the grouping and administration method in the 4 dose groups of Ansofaxine Hydrochloride Extended Release Tablet and placebo group.

Table1 Grouping and administration method

| **DURATION OF TREATMENT** | **Dose group** | **Drug administered** | **Method of Administration** |
| --- | --- | --- | --- |
| Wash-out period | -- | △△ | **Administration method**  Once per day, at a relatively fixed time in the morning, under fasted state or after meal.  **Precautions:**   1. During the washoutperiod, the subjectstook 2 tablets on the same aluminum plastic plate. 2. During the treatment period, thesubjects took 4 tablets on the same aluminum plastic plate. 3. The investigational drug should beswallowed whole and should not be crushed or chewed or taken after dissolving. 4. Ask the subjectsabout the habit oftaking this medication (fasting orpostprandial) and remind the subjects to beconsistent throughout the trial. |
| Treatment Period | Ansofaxine Hydrochloride Extended Release Tablet 40mg group | ▲△△△ |  |
|  | Ansofaxine Hydrochloride Extended Release Tablet 80mg group | ▲▲△△ |  |
|  | Ansofaxine Hydrochloride Extended Release Tablet 120mg group | ▲▲▲△ |  |
|  | Ansofaxine Hydrochloride Extended Release Tablet 160mg group | ▲▲▲▲ |  |
|  | Placebo group | △△△△ |  |

▲ Ansofaxine Hydrochloride Extended Release Tablet △Placebo

## Dose adjustment

Change the dosage is not allowed in the clinical trial. The subjects may be suspended if subjects experience adverse events and was agreed to by the investigator, the subjects will get other treatment based on inverstigator’s judgement.

## Drug Accountability

At each visit, investigators should record the number of all the drugs received, taken and returned by subjects carefully, as to make a judgment on the subject’s compliance with the medication and determine if the subject can continue to participate in this study. If the subject is taking the drug on a regular basis as required in the protocol at each evaluation, and has taken 80%～120% of the dosage to be taken, the subject will be considered to have a good compliance.

## Drug storage

Study drug must be stored at room temperature in sealed package. Study drug will be in charged by the pharmacist, subjects will be assigned to dose regimens, will receive their assigned dose and administrate, only subjects who has enrolled in the trial will be administrate the study drug. Receive and return the drug from subjects will be documented appropriately. The investigator or its authorized personnel will check and record the study drug’s appearance, storage condition, usage report, expire time, and temperature log for the storage of study drug periodically.

## Evaluation of compliance with medication

Subjects need to return the package of used drugs and remaining drugs in the package at the follow-up visit. Investigators or designated personnel will check the number of drugs returned, calculate the number of drugs used and compare with the number that should be used. Lost or unreturned drugs need to be recorded.

Compliance will be calculated, as follows:

Compliance＝number of tablets taken/number of tablets expected to be taken × 100%.

If the percent compliance calculated using the above formula is less than 80% or greater than 120%, the subject will be considered as incompliance with the quantitative administration.

The reason should be found out and recorded in case of poor compliance.

## Concomitant medicationand therapy

Data on the previous medications within 30 days, non-drug therapies within 90 days prior to screening and concurrent therapies throughout the study will be collected.

### Contraindicated drugs and therapy

The following concomitant medications and concurrent therapies are not allowed to be used throughout the study:

1. Any antipsychotic drug, antidepressant, antianxietic, mood stabilizer and traditional Chinese medicine preparation with the function of relieving depression and calming nerves (including health products);
2. Electroconvulsive therapy;
3. Acupuncture and moxibustion, or other traditional Chinese medicine treatment;
4. Systematic psychotherapy;
5. Transcranial magnetic stimulation therapy.

### Permitted concomitant medications and therapies

1. For the patients with severe insomnia, Zaleplon, Zopiclone, Eszopiclone, Zolpidem can be used, at the dose not exceeding the upper limit specified in the package insert (maximum recommended dose), before bedtime, for no more than two weeks continuously during the study.
2. Symptomatic non-systematic psychotherapy is allowed.
3. Concomitant medication for treatment of somatic diseases is allowed during the study, and it would be best to keep the type and dose of the medication unchanged during treatment.

The generic name (or name of other therapy), dosage, frequency and duration of the drugs or other therapies that must be continued for concurrent diseases must be recorded in the case report form.

# Follow-up procedure

The follow-up schedule and content are seen in Figure 1 in this study, the patients may be followed up for safety at any time during that period.

Fig. 1 Study flow chart

|  | **Screening/wash-out period^1^** | **Baseline** | **Double-blind treatment period (6 weeks)** | | | |
| --- | --- | --- | --- | --- | --- | --- |
| **Visit** | **Visit 1**  **-14 ~ -1 d** | **Visit 2**  **0d** | **Visit 3**  **7 ± 1d** | **Visit 4**  **14 ± 1d** | **Visit 5**  **28 ± 3d** | **Visit 6/ at termination**^2^  **42 ± 3d** |
| Informed consent | × |  |  |  |  |  |
| Inclusion and exclusion criteria | × | × |  |  |  |  |
| General information | × |  |  |  |  |  |
| Medical history and psychiatric symptoms | × |  |  |  |  |  |
| DSM-IV-TR diagnostic criteria | × |  |  |  |  |  |
| Randomization |  | × |  |  |  |  |
| HAM-D_17_ | × | × | × | × | × | × |
| HAMA |  | × | × | × | × | × |
| CGI-S | × | × | × | × | × | × |
| CGI-I |  |  | × | × | × | × |
| VAS-PI |  | × | × | × | × | × |
| Physical examination | × |  |  |  |  | × |
| Vital signs^3^, body weight | × | × | × | × | × | × |
| Laboratory test^4^ | × |  |  | × |  | × |
| FT3, FT4,TSH | × |  |  |  |  |  |
| HbsAg, anti-HCV | × |  |  |  |  |  |
| Urine pregnancy test^5^ | × | × |  |  |  | × |
| Urine drug screening^6^ | × |  |  |  |  |  |
| ECG | × | × |  | × |  | × |
| AE record(s) | × | × | × | × | × | × |
| Recording of concomitant medication | × | × | × | × | × | × |
| Record of drug dispensation and administration | ×^7^ | × | × | × | × | × |
| Record of end of study |  |  |  |  |  | × |

Note:

1. Screening/wash-out period: on Day -14~-1, all the subjects will be dispensed with the drugs for wash-out period on Day -8 and enter the 7-day wash-out period (-7 ± 1d).
2. For the subjects who terminate the study prematurely, they will complete the last visit in accordance with the evaluation content at the end of Week 6 (Day 42).
3. Respiratory frequency, axillary temperature, pulse rate and blood pressure in supine and orthostatic position need to be measured (please see 8.2.1 for relevant description).
4. Laboratory examination: the complete blood cell count includes white blood cell count, lymphocyte count, neutrophil count, eosinophil count, basophil count, monocyte count, red blood cell count, hemoglobin, hematocrit, platelet count; routine urinalysis includes white blood cell, PH, nitrite, protein, glucose, ketone body, urobilinogen, bilirubin, red blood cell, specific gravity; serum chemistry includes alanine aminotransferase, aspartate aminotransferase, total protein, albumin, alkaline phosphatase, γ- glutamyl transpeptidase, total bilirubin, direct bilirubin, urea nitrogen, creatinine, uric acid, sodium, potassium, chlorine, calcium, phosphorus, total cholesterol, triglyceride, high-density lipoprotein cholesterol, low-density lipoprotein cholesterol, fasting blood glucose, lactate dehydrogenase, creatine kinase, creatine kinase isoenzyme; serology includes testosterone and prolactin.
5. It is applicable for the women of childbearing potential.
6. Urine drug screening test: morphine, methamphetamine, methadone, Phencyclidine, Tetrahydrocannabinolic acid, amphetamine, cocaine, Barbiturates, Benzodiazepines, tricyclic antidepressants.
7. Drugs in wash-out period: dispensed on Day -8 and used from Day -7.

Abbreviations: HAM-D_17_＝ Hamilton Depression Scale – 17; HAMA= Hamilton Anxiety Scale; CGI-S = Clinical Global Impression scale – severity; CGI-I = Clinical Global Impression scale – overall improvement; VAS-PI = Visual analogue scale - pain intensity; FT3 = free triiodothyronine; FT4 = free thyroxine; TSH = thyroid stimulating hormone; HbsAg = hepatitis B surface antigen; anti-HCV = hepatitis C antibody

## Screening/wash-out period

**Visit 1— — Day -14 ~ -1**

The objective of this visit is to investigate if the patient is suitable to be enrolled in this study. During this period, investigator will carry out the following on the potential candidates:

- Signed informed consent;
- Demographics: including nationality, date of birth, sex, etc.;
- History inquiry, including the occurrence of disease, therapies prior to screening and therapies and medications for other concurrent diseases;
- Major depressive disorder will be confirmed in accordance with DSM-IV-TR criteria;
- Evaluation: HAM-D_17_, CGI-S;
- Physical examination: including general condtion, skin and mucosa, lymph node, the five sense organs, head and neck, thyroid gland, heart, lungs, abdomen, spine and four limbs, motor system and nervous system, including height and weight;
- Vital signs: blood pressure and pulse rate (in supine and orthostatic position), temperature, respiratory frequency;
- 12-Lead ECG;
- Laboratory safety examinations: including complete blood cell count, routine urinalysis, serum chemistry and serology;
- Screening tests: HBsAg, anti-HCV; FT3, FT4, TSH; urine drug screening;
- Urine Pregnancy test (for women of childbearing potential only);
- Recording of concomitant medication;
- Evaluation of adverse event.

All the subjects meeting the inclusion criteria and not meeting the exclusion criteria will enter the one-week placebo wash-out period. Investigators will dispense the drugs for wash-out period on Day -8 and describe the administration method and precautions in detail. Subjects will receive the drugs on the following day (Day -7) for consecutive 7 days, the acceptable time window for the wash-out period is -7 ± 1 day. A follow-up visit after 7 days will be scheduled. If the patient has any question on this study or abnormal condition during this period, he/she can contact the study personnel at any time.

## Baseline

**Visit 2 — — Day 0**

- Enquiry about the occurrence of adverse event;
- Enquiry about concomitant medications;
- Evaluation: HAM-D_17_、HAMA、CGI-S、VAS-PI；
- Vital signs: blood pressure and pulse rate (in supine and orthostatic position), temperature, respiratory frequency;
- Weight;
- 12-Lead ECG;
- Urine Pregnancy test (for women of childbearing potential only);
- The residual study drug in wash-out period will be recoverd, packaged and recorded;
- Whether the subject meets the inclusion/exclusion criteria will be re-evaluated, those meeting the inclusion/exclusion criteria will be randomized to each Ansofaxine dose group or placebo group, and dispensed the study drug. Subjects will receive the study drug on the following day (Day 1);
- The next visit will be scheduled, and the subjects will be remined to bring residual study drug and package back at that visit.

## Double-blind treatment period

There will be window period of±(1~3) days for all the visits in double-blind treatment period, as to allow for minor changes in subject's schedule, however, subjects should be followed up on the scheduled date as far as possible. When arranging subsequent visits, it needs to be ensured that the total duration of treatment is the duration of treatment in the study protocol, i.e., the subsequent visit can not be scheduled on the pervious visit but on the baseline visit.

### Visit 3 — — At the end of Week 1 (7±1 day)

- Enquiry about the occurrence of adverse event;
- Enquiry about concomitant medications;
- Evaluation: HAM-D_17_、HAMA、CGI-S、CGI-I、VAS-PI;
- Vital signs: blood pressure and pulse rate (in supine and orthostatic position), temperature, respiratory frequency;
- Weight;
- The residual study drug will be recoverd, packaged and recorded, the study drug will be dispensed;
- The next visit will be scheduled, and the subjects will be remined to bring residual study drug and package back at that visit.

### Visit 4 — — At the end of Week 2(14±1 day)

- Enquiry about the occurrence of adverse event;
- Enquiry about concomitant medications;
- Evaluation: HAM-D_17_、HAMA、CGI-S、CGI-I、VAS-PI;
- Vital signs: blood pressure and pulse rate (in supine and orthostatic position), temperature, respiratory frequency;
- Weight;
- Laboratory safety examinations: including complete blood cell count, routine urinalysis, serum chemistry and serology;
- 12-Lead ECG;
- The residual study drug will be recoverd, packaged and recorded, the study drug will be dispensed;
- The next visit will be scheduled, and the subjects will be remined to bring residual study drug and package back at that visit.

### Visit 5 — — At the end of Week 4 (28±3 days)

- Enquiry about the occurrence of adverse event;
- Enquiry about concomitant medications;
- Evaluation: HAM-D_17_、HAMA、CGI-S、CGI-I、VAS-PI;
- Vital signs: blood pressure and pulse rate (in supine and orthostatic position), temperature, respiratory frequency;
- Weight;
- The residual study drug will be recoverd, packaged and recorded, the study drug will be dispensed;
- The next visit will be scheduled, and the subjects will be remined to bring residual study drug and package back at that visit.

### Visit 6 — — At the end of Week 6 (42±3 days)

- Enquiry about the occurrence of adverse event;
- Enquiry about concomitant medications;
- Evaluation: HAM-D_17_、HAMA、CGI-S、CGI-I、VAS-PI;
- Physical examination;
- Vital signs: blood pressure and pulse rate (in supine and orthostatic position), temperature, respiratory frequency;
- Weight;
- Laboratory examinations: including complete blood cell count, routine urinalysis, serum chemistry and serology;
- 12-Lead ECG;
- Urine pregnancy test (for women of childbearing potential);
- The residual study drug will be recovered, packaged and recorded;

### Premature withdrawal visit

Subjects can withdraw from the study at any time according to their own wishes, investigators or the sponsor can request the subject to withdraw from the study at any time for safety reason, or as the subject can not comply with the visit schedule required in the study protocol or procedure at the study site. If possible, the last visit of the subject should be completed according to the evaluation contents at the end of Week 6. The reason for discontinuation should be recorded in detail in the source document and CRF.

# Evaluation variable(s)

## Efficacy variables

**Primary variables:**

Changes from baseline in the total score of 17 items of Hamilton Depression Scale (HAM-D_17_) at the end of treatment.

**Secondary variables:**

- Change in the Clinical Global Impression Scale-Improvement (CGI-I) score at the end of treatment;
- Change from baseline in the Clinical Global Impression Scale-Severity (CGI-S) score at the end of treatment;
- Change from baseline in the Hamilton Anxiety Scale (HAMA) at the end of treatment;
- Change from baseline in the Visual Analog Scale of Pain Intensity (VAS-PI) score at the end of treatment;
- Change from baseline in HAM-D_17_ factor scores at the end of treatment;
- HAM-D_17_ response rate (≥ 50% reduction from baseline in HAM-D_17_ total score);
- HAM-D_17_ remission rate ( HAM-D17 total score ≤7).

## Safety Indicators

- Adverse event
- Vital signs, physical examination, laboratory tests (hematology, urinalysis, blood chemistry test and serological test), 12-lead ECG findings

Patients will be followed up by investigators according to the scheduled procedure, vital signs, laboratory examinations and adverse events will be checked and recorded. Unexplainable laboratory outliers should be re-measured immediately, until they are recovered to normal and/or can be well explained.

All the adverse events occurred during the study should be recorded in CRF. Evaluation of adverse event includes classification, grade, relationship with the drug, therapeutic measures and outcome.

For the subjects prematurely withdrawn, every effort should be made to determine the reason for the withdrawal, record the time of withdrawal from the study precisely and complete all the examinations at the last visit.

### Vital signs, weight

Vital signs and body weight should be measured at screening, baseline, each visit and premature termination of the study.

Weight (kg): usual clothes and shoes can be wore, outer clothing can not be worn.

Tempreture (℃): axillary

Blood pressure (mmHg): in supine and orthostatic positions

Pulse rate (beat/min): in supine and orthostatic positions

Respiratory frequency (time/min): in sedentary state

Blood pressure and pulse rate measurement procedure

Prior to measurement of blood pressure, subjects should be required to abstrain from smoking or caffeine-containing beverages for at least two hours. A mercury sphygmomanometer or electronic sphygmomanometer with a suitable circumference of the subject's arm should be used to measure blood pressure. The blood pressure will be measured through the same arm by the same study personnel as far as possible throughout the study. Subjects should rest for at least two minutes prior to measurement of blood pressure (BP) at each visit. 2 consecutive BP readings in supine position will be determined at an interval of two minutes. The pulse rate in supine position will be determined between the first and second measurement of BP in supine position, because this can make sure that pulse rate can be determined within the time limit of two minutes.

The subject will get up and stand up after the 2nd measurement of blood pressure in supine position. After standing for one minute, the 2nd measurement of orthostatic BP will be performed (at an interval of 2 minutes). The pulse rate in orthostatic position will be determined between the first and second measurement of BP in orthostatic position, because this can make sure that pulse rate can be determined within the time limit of two minutes. The systolic blood pressure and diastolic blood pressure will be determined using Korotkoff Sound I and V (vanishing sound). The utmost effort will be made to acquire vital sign measurements at the same time point at each visit, and the vital signs should be measured by the same person as far as possible.

### Physical examination

It will be carried out at screening, visit 6 and premature termination of the study, including general condition, skin and mucosa, lymph node, the five sense organs, head and neck, thyroid gland, heart, lungs, abdomen, spine and four limbs, motor system and nervous system, height will be measured only at screening.

### 12-Lead ECG

12-lead ECG will be performed at screening, baseline, visit 4 and 6 or premature termination, the subjects should rest for at least 5 minutes prior to each measurement.

### Laboratory tests

1. Laboratory safety examinations will be performed at screening, visit 4 and 6 or end of the study, including:

- Complete blood cell count: white blood cell count, lymphocyte count, neutrophil count, eosinophil count, basophil count, monocyte count, red blood cell count, hemoglobin, hematocrit, and platelet count.
- Routine urinalysis: white blood cell, PH, nitrite, protein, glucose, ketone body, urobilinogen, bilirubin, red blood cell, specific gravity.
- Serum chemistry: alanine aminotransferase, aspartate aminotransferase, total protein, albumin, alkaline phosphatase, γ-glutamyl transpeptidase, total bilirubin, direct bilirubin, urea nitrogen, creatinine, uric acid, sodium, potassium, chlorine, calcium, phosphorus, total cholesterol, triglyceride, high-density lipoprotein cholesterol, low-density lipoprotein cholesterol, fasting blood glucose, lactate dehydrogenase, creatine kinase, creatine kinase isoenzyme.
- Serology: testosterone, prolactin.

1. Laboratory examination screening variable, only performed at screening, including:

- Free triiodothyronine, free thyroxine, thyroid-stimulating hormone
- Hepatitis B surface antigen and hepatitis C antibody
- Urine drug screening test: morphine, methamphetamine, methadone, Phencyclidine, Tetrahydrocannabinolic acid, amphetamine, cocaine, Barbiturates, Benzodiazepines, tricyclic antidepressants

1. Urine pregnancy test will be performed for women of childbearing potential at screening, baseline, visit 6 and end of treatment.

All the abnormal laboratory examination items of clinical significance will be re-measured after start dose of study drug, until they are recovered to normal, or baseline level, or stable. If the laboratory examination value is not recovered to normal, or baseline level or stable within a reasonable time limit, relevant reason should be found out and the sponsor will be informed.

### Risks and precautions during the study

1. Excerbation of clinical symptoms and suicide

For patients at any age with major depressive disorder, their disease may be possibly exacerbated, regardless of the administration of antidepressants, and they are likely to have suicidal ideation and suicidal behavior as well as abnormal change of behavior, and this risk may continue until the condition is significantly relieve. Depression is known to be associated with some mental disorders and suicide risk, and these mental disorders themselves are the strongest signs of suicide.

For all the subjects receiving the study drug (Ansofaxine Hydrochloride Extended Release Tablet or placebo) for treatment, exacerbation of their clinical symptoms, suicidal tendency and abnormal change in behavior should be closely observed, in particular in the initial stage of drug treatment.

When antidepressants are used for treatment of adult and pediatric patients with major depressive disorder, other psychotic or non-psychotic disorders, the following symptoms may appear: anxiety, agitation, panic attack, insomnia, irritability, hostility, aggression, impulsiveness, restlessness (psychomotor restlessness), hypomania and mania. Although the correlation of the occurrence of the above symptoms with exacerbation of depression and/or suicidal impulse has not been identified, these symptoms may be the foreboding of suicide.

For the subjects with persistent exacerbation of depressive symptoms, suicide or signs associated with it, discontinuation of study drug should be considered, in particular for those whose symptoms are severe, occur suddenly or newly appear.

In this study, the subjects with suicide risk should be excluded.

1. Prolonged QT interval

The in vitro study showed that Ansofaxine Hydrochloride could inhibit the potassium channel coded by hERG, however, no prolonged QT interval was seen overall in the animal study, nor clinically significant prolongation of QT interval was seen in the phase I clinical trial on tolerability. In order to ensure subject’s safety, subjects with clinically significant abnormality on ECG (in particular prolonged QTc interval) should be excluded, and will be monitored through ECG during the study.

1. Blood Pressure

It was observed in the pharmacological study on cardiovascular safety in Cynomolgus monkeys that this product could induce elevated blood pressure in the animals, however, no clinically significant change was seen in the blood pressure in the phase I clinical trial on tolerability. In order to ensure subject’s safety, effect of the drug on blood pressure should be noted during the clinical study. In this study, hypertensive patients with poor control of blood pressure should be excluded, and will be monitored through blood pressure in supine and orthostatic blood pressure at each visit.

# Adverse Events Reporting

Adverse event (AE) is defined as any unfavorable medical event that occurs in the subject who receives study drug in clinical trial, which does not necessarily have a causal relationship with the drug. In this study, adverse events (serious adverse event and non-serious adverse event) from the signature of informed consent form to the last visit will be recorded.

## AE record(s)

The adverse event record form should be filled in truthfully during the study. Its correlation with the study drug will be evaluated based on comprehensive consideration of complications and concomitant medications, through detailed record of its type, intensity, time of occurrence, duration, therapeutic measure and treatment.

All the physical examination and laboratory examination findings required in the study protocol should be recorded in the subject’s CRF. The physical examination and laboratory examination findings after administration will be compared with that prior to administration, the change must be evaluated as to judge if it meets the definition of adverse event if it is indicative of exacerbation of clinical status, and all the changes determined to meet the definition of adverse event will be recorded in Section Adverse event in CRF.

The medical document on adverse event should be recorded in the original document, including the laboratory examination report form.

## Criteria for severity assessment of adverse events

When filling in the Adverse event form in CRF, investigators will describe the intensity of adverse event with mild, moderate and severe. In order to unify the criteria, the intensity of event will be graded as below:

Mild: no interference with subject’s daily activities.

Moderate: interference to some extent with subject's daily activities.

Severe: serious interference with subject’s daily activities.

Attention should be paid not to confound severe adverse event with serious adverse event: severe is one category to measure the severity of an event, adverse event and serious adverse event can both be assessed as severe, all the events meeting the definition of serious adverse event should be listed as serious adverse event.

## Assessment criteria for the correlation between AEs and investigational product

The correlation with the study drug should be evaluated for all the adverse events, and the incidence will be calculated for definitely related,positively related, probably related and possibly related adverse reactions.

| Incidence of adverse reactions = | Number of subjects with at least one adverse reaction | × 100% |
| --- | --- | --- |
|  | Total number of subjects for evaluation of safety |  |

The judgment criteria for the correlation between adverse event and investigational product are seen in Table 2

Table 2 Assessment criteria for the correlation between AEs and investigational product

| Indicators | Results | | | | |
| --- | --- | --- | --- | --- | --- |
|  | Definitely related | Probably related | Possibly related | Possibly not related | Unrelated |
| The time of occurrence of the event coincides with the time of administration | ＋ | ＋ | ＋ | ＋ | ＋ |
| The adverse event is related with the known adverse reaction of the drug | ＋ | ＋ | ＋ | － | － |
| The adverse event can be explained with other reasons | － | － | ± | ± | ＋ |
| The adverse event is resolved following discontinuation of the drug | ＋ | ＋ | ± | ± | － |
| The adverse event reappear after dose resumption | ＋ | ？ | ？ | ？ | － |

## Treatment and follow-up of adverse event

Adverse event should be actively treated, regardless of the causal relationship between the event and investigational product. Acceptable clinical therapeutic measures should be taken for treatment of the patient experiencing adverse event. If it is absolutely necessary to use the medical measures rejected in the study project, it should be discussed with the sponsor if the patient needs to be withdrawn from the study.

Adverse event will be followed up not only in treatment period, for some subjects, the adverse event related with the investigational product that is still present after end of the study should be followed up on an ongoing basis until any one of the following conditions is met:

1. The event is resolved;
2. The event is stable;
3. The event returns to baseline level;
4. The event can be attributed to a drug other than the investigational product or unrelated with the study behavior;
5. More information is unlikely to be obtained (the patient refuses to provide more information, or as proven by the evidence, the patient is still lost of follow-up in despite of utmost effort).

## Serious Adverse Event

### Serious adverse event definition

Serious adverse event (SAE) is defined as the following important medical event occurred at any dose:

1. Requiring hospitalization or prolongation of existing hospitalization;
2. Disability;
3. Affecting working capacity;
4. Being life-threatening or death;
5. Leading to congenital deformity;
6. An important medical event (for example, it may affect the subject or require medication/surgery to prevent the above outcomes).

Some events requiring hospitalization or prolongation of existing hospitalization may not be serious adverse events, including hospitalization for other reasons rather than adverse event, hospitalization for scheduled surgery prior to the study or the purpose of other treatment or examination.

When determining whether an event is an important medical event, scientific medical judgment needs to be made. An important medical event may not be immediately life-threatening and/or may not lead to death or hospitalization. However, if this event is determined to possibly endanger the subject or requires interventional measures to prevent one of the above outcomes of one adverse event, this important medical event should be reported as serious adverse event.

### Management and record of serious adverse event

In case of any serious adverse event occurred during the study, regardless of its relationship with the study and investigational product, investigators should make a full evaluation of the event immediately, complete serious adverse event report form and fax to Shandong Luye Pharma Co., Ltd. within 24 hours, whilst reporting to the ethics committee, CFDA, Shandong Food and Drug Administration and the provincial Food and Drug Administration where the study unit is located within 24 hours.

## Pregnancy

The clinical study pregnancy form must be used to report any pregnancy occurred during participation in the study. In order to ensure subject’s safety, each pregnancy must be reported to Shandong Luye Pharma Co., Ltd. within two weeks after awareness of it. The pregnancy must be followed up, as to determine the prognosis and state of the mother and infant (including early termination of pregnancy). Complications of pregnancy and targeted termination of pregnancy for medical reasons must be reported as AE or SAE. Spontaneous abortion must be reported as SAE.

If the subject has any pregnancy related SAE after completion of the study, which is learnt by the investigator and judged by the investigator as possibly related with the investigational product, it must be reported to Shandong Luye Pharma Co., Ltd. immediately.

In addition, if any female partner of male subject is pregnant during participation of the male subject in this study, the investigator must try to collect the data on the pregnancy. As described above, data on pregnancy must be reported to Shandong Luye Pharma Co., Ltd.

# Unblinding

MagMin central randomization system has the function of unblinding at the same time. Prior to the start of the study, the principal investigator at each site will be allocated one emergency ublinding code by system administrator, as to be used in case of emergency blinding (one more code can be distributed successively after use). After end of the study, the unblinding and randomization will be submitted simultaneously for archival. It is specified for unblinding individual cases in case of emergency that the responsible investigator at sub-site will report to the monitor, sponsor and principal investigator to determine emergency unblinding, when serious adverse event or death occurs, or urgent rescue is needed.

It is specified for the preservation of blind base that Level I is the group corresponding to drug numbers and Level II is the treatment corresponding to the two groups. Two levels of blind base will be sealed separately, in triplicate, and kept by the leading unit and the sponsor, respectively, and for submission.

It is specified for unblinding that after all the study data have been verified and locked, the statistical plan will be discussed by the principal investigator, statisticians and the sponsor jointly, and the first unblinding will be carried out, the three parties will sign on the blind base.

Upon unblinding, any modification to the database needs to be approved by the principal investigator, statisticians, data administrator and the sponsor in a written form.

# Data managementand statistical analysis

## Data management

### Data collection and transfer

Investigators must ensure the data are true, complete and accurate.

Except the data collected directly by computer or automatic instrument, all the data generated from actual study need to be recorded in the form, arranged and bound at any time. All the data records are dated and signed by the recorder.

Investigators will fill in the CRF and sign in a timely, complete and accurate manner in accordance with the subject’s orginal condition, the CRF must be completed for all the included cases. All the items on CRF need to be filled in and must not be blank or missed; investigators can only underline, note new data on the side, provide the reason, sign and date when doing any change, and can not wipe or cover the original record. Laboratory examination items are complete.

### Data Monitoring

Monitors wil monitor the whole course of the study as required in the protocol. Monitors will review the original data and CRF, as to ensure the clinical study data are recorded in a timely, accurate, standardized and complete manner, ask investigators to correct in time if there is any error or omission. The original record should be clearly visible with investigator’s signature and date during the modification.

### Data transfer

The CRF verified by monitors should be signed by monitors, and submitted to data administrator in time. The transfer of completed CRF across investigators, monitors and data administrator should be documented, and signed and dated by the handover person.

### Data entry and modification

Data entry and management will be the responsibility of data administrator at the statistical unit. After receipt of the first copy of CRF, data administrator shall establish one dedicated database for data entry and management. The database naming should be standardized, easy to read and easy to search. Data need to be entered and proofread independently in duplicate.

### Data Lock

The data will be locked upon confirmation by the principle investigator, the sponsor, statistical analysis personnel and monitors. The data file locked shall no more be changed. The questions found after lock of database can be modified in the statistical analysis program upon confirmation.

### Data archival

After completion of data entry and verification as required, CRF should be filed for preservation in the order of numbering, electronic data files, including database, inspection procedure, analysis procedure, analysis result and coding bood, shall be backed up on different recording media for proper storage.

## Statistical analyses

After study protocol is finalized, the statistical analysis plan will be generated by professional statisticians through the discussion with principal investigator.

### Analysis set of clinical trial

The analysis set defined for this study is as below:

1. Full analysis set (FAS)

It includes all the randomized subjects who were administrated at least 1 dose of double-blind study drug and had primary efficacy evaluation at baseline and at least one primary efficacy evaluation after the first dose of double-blind treatment. Unless otherwise specified, LOCF (last observation carried forward) will be used for the missing value imputation in the FAS.

FAS is the primary analysis data set for this trial.

1. Per protocol set (PPS)

It includes all the subjects who completed visit 6 and have no major protocol deviation.

1. Safety set (SS)

It includes all the subjects who were randomized and administrated at least one dose of the double-blind study drug. Subjects who were dispensed study drug but did not administrate any study drug will not included in SS.

### Analysis of Efficacy

The final treatment evaluation will be the primary evaluation for all the primary and secondary efficacy variables. The double-blind treatment starts from the first dose of study drug, until the last dose of study drug. PPS and FAS are used for the analysis of efficacy. The analysis of PPS is based on the observed data. Unless otherwise specified, LOCF (last observation carried forward) and the observed data will be used for analysis of FAS.

The missing items of observations on the efficacy scale will be processed as described below: if up to 20% of the items are missing on one scale, the total score can be derived by multiplying the average of the available items by the total number of items. If more than 20% of the items are missing, the total score on this scale will be considered as missing. The missing total scores will be imputed using the LOCF method. For observed case (OC) analysis, the missing total scores will not be imputed. The baseline score will not be used to carry forward.

In the statistical model using study site as one independent variable the site with small number of subjects may be pooled. The anlaysis will be performed after the sites were pooled. The rules of pooled site will be provided in the statistical analysis plan, if necessary. In addition, this study is one exploratory analysis, and no adjustment of multiple tests will be carried out.

1. Analysis of the primary endpoints

The primary endpoint is the change in the total score of HAM-D_17_ scale from baseline at the final treatment evaluation, and the statistics including the number of subjects, mean, standard deviation, median, minimum and maximum will be calculated.

This is one dose-effect exploratory study, α for type I error is set as 0.1.

In the exploratory analysis of efficacy in each dose group, the mean plots for the efficacy endpointover time will be created firstly (visit time as the x axis, the mean HAM-D_17_ scale in each group as the y axis), as to observe the trend of change in the therapeutic effect over time in each dose group, and repeated measure analysis of variance (time as intragroup factor, treatment group as intergroup factor) will be used to explore the statistical significance among the doses; secondly, the mean plots for the efficacy endpoint among dose will be created (dose as the x axis, the mean HAM-D_17_ scale at the end of Week 6 as the y axis), and ANCOVA model is used to compare the change in the total score of HAM-D_17_ scale from baseline at the end of Week 6 between treatment groups, using total score of HAM-D_17_ at baseline as covariate, treatment group and site as fixed effect, as to explore of the relationship between efficacy and dose (linear growth, exponential growth or "S" growth).

1. Analysis of the secondary endpoints

The scores on each scale will be summarized using the number of subjects, mean, standard deviation, median, minimum, maximum and interquartile range by visit; the number of subjects and percentage will be used for the response rate and remission rate by visit. The analysis method for each secondary endpoint variable is as below:

CGI-I and CGI-S scale: ANOVA will be used for CGI-I, the model will be include treatment group and site as factors; ANCOVA will be used for CGI-S, the model considers the change from baseline as dependent variable, treatment group and site as fixed fators, and baseline of CGI-S as covariate variable.

HAMA and VAS-PI: ANCOVA model will be used to evaluate the change from baseline in the total score of HAMA scales, psychological anxiety score on HAMA scale, somatic anxiety score on HAMA scale and change in each subcomponent of VAS-PI at each time point, the model factors are treatment group and site, the corresponding baseline score is the covariate.

HAM-D_17_ response rate: HAM-D_17_ responder is defined as the subject with HAM-D_17_ score reduced by ≥50% from baseline. Cochran-Maenszel-Haensel test (corrected by site) will be used to analyze the response rate at each time point.

HAM-D_17_ remission rate: HAM-D_17_ remission rate is defined as the percentage of subjects with HAM-D_17_total score ≤7. Cochran-Haenszel test (corrected by site) will be used to analyze HAM-D_17_ remission rate at each time point.

### Safety Analysis

The descriptive method will be used for the safety evaluation, and safety variables include adverse event, dose interruption for adverse event, vital signs, laboratory measurement (complete blood cell count, routine urinalysis and serum chemistry), standard 12-lead ECG and physical examination findings.

Type, severity, frequency and the relationship with the investigational product will be tabulated for description for all the adverse events occurred during the study. The subjects that occurred the adverse events leading to withdraw from study and serious adverse events will be particularly noted. Adverse events will be coded using the Medical Dictionary for Regulatory Activities (MedDRA). The shift table will be used for the lab test. Abnormal test items with clinical significance need to be listed.

### Interim Analysis

No interim analysis is planned in this study.

### Data monitoring committee

This study will not use a Data Monitoring Committee.

# Quality control and assurance

1. This clinical study will be carried out in CFDA certified drug clinical trial institutions. The study personnel have the professional knowledge and experience required for this clinical study and are relatively stable.
2. Prior to the start of the study, all the investigators participating in this study will be organized to study the protocol carefully, as to be familiar with the study methods and procedures, and implement each technical indicator.
3. Prior to the start of the study, relevant scale assessor will be trained on the consistency of scale evaluation.
4. Subject’s cooperation is very important. They will be asked to understand the significance of this study fully, cooperate with treatment and observation actively as required in the study protocol.
5. Other drugs and therapies affecting judgment of results are contraindicated during the study.
6. The laboratory has established standard operating procedure and quality control procedure for laboratory observational variables.
7. The sponsor and study unit will establish quality control and quality assurance systems, respectively.
8. The sponsor will appoint monitors, as to make sure the subject’s rights are guaranteed during the study, the study record and reported data are authentic, accurate and complete without error, and the study complies with the approved protocol, GCP and relevant regulations.
9. During the study, monitors will monitor the progress and quality of the study on a regular basis, as to ensure the study is carried out according to the protocol, and verify CRF, as to ensure consistency with the original data.
10. The study drug will be maintained by the person in charge of drug management, locked in a specially assigned counter and kept at room temperature. The residual drugs will be stored separately and registered for the amount, and returned to the sponsor centrally at the end of the study.
11. Archive keeping, data processing and correction of relevant testing instrument need to be managed by specially assigned person.

# Ethical requirements

## Good Clinical Practice

This clinical study will be carried out in accordance with the Declaration of Helsinki and GCP requirement. The study protocol will be conducted only upon approval by the ethics committee. Investigators will make sure that this clinical study is conducted in accordance with the laws, regulations, scientific and ethical standards of the People's Republic of China on medical research.

## Informed consent

Subjects need to sign a written informed consent form prior to enrollment in the study (screening or any other activity related with the study). Investigators will explain the materials related with the study drug to each subject sufficiently, including the property, objective, procedure, observational variables, examinations, possible risks and benefits, as well as possible adverse reactions subject’s rights and duties, and provide sufficient time for subjects to consider the content related with the study, as to make the decision on whether to participate in it. Subject’s personal information will be strictly confidential, unless required by laws, subject’s identity, privacy and test results will not be revealed.

## Early termination of the trial

If the frequency and severity of adverse event imply potential risk of the study drug to human health, this clinical study will be terminated. The sponsor, ethics committee and relevant regulatory authorities should be reported prior to the termination.

# Summary Report

Enrollment and observation of all the cases are expected to be completed in 12 months in this clinical study. Upon completion of observation of all the cases, biostatisticians will carry out statistical analysis of all the data and issue the statistical analysis report, provide to the leading unit of this study to issue summary report.

# Amendment to study protocol

In case amendment to the study protocol is found during the study, it needs to be discussed by the principal investigator and the sponsor jointly, and approved through signature of both parties. After amendment to the protocol, it can be carried out only upon putting on file/approval by the ethics committee. If any important new material involving the investigational product is found, the informed consent form needs to be modified in a written form and submitted to the ethics committee for review, and subject’s consent must be obtained again.

# Publication

Investigators can publish or disclose the results of the clinical study after end of the clinical study, however, a prior writtern approval from the sponsor is needed. For multi-center clinical study, the principal investigator must make sure that the data at one single study site can not be published or disclosed in advance prior to publication or disclosure of the complete study results.

The sponsor or its representative will reserve the right to review the first draft before publication or disclosure of the study results. This is not to restrict or prevent investigators from publishing or disclosing clinical study results, but to protect intellectual property information owned by the sponsor or its representative, and allow the sponsor or its representative to provide advice on the clinical study results planned to be published or disclosed based on the information not yet available to investigators.

# Archival of materials

In order to ensure the evaluation and supervision of CFDA and the sponsor for the clinical study, investigators should agree to keep all the study materials, including the original record during hospitalization, informed consent form, CRF and detailed record of drug dispensation, until 5 years after end of the clinical study. Prior to destruction of any clinical study record, the study personnel must inform Shandong Luye Pharma Co., Ltd. If the study personnel hope to remove the study records to other sites, Shandong Luye Pharma Co., Ltd. must be informed in advance. If the study personnel can not guarantee that some or all of the documents can meet the requirements for archival at the study institution, special arrangements must be made between study personnel and Shandong Luye Pharma Co., Ltd., these documents will be placed in a sealed container in another place, so that they can be returned to the study personnel intact when required for statutory audit. The original documents that must be retained in order to continue to pay attention to the subjects can be appropriately copied, and the copies will be kept outside of the study institution.

# References

1. State Food and drug administration. Technical guidelines for clinical trials of antidepressant drugs (Draft) 2013.
2. Wyeth Pharmaceuticals Inc. High lights of prescribing information: prescribing information for Pristiq (desvenlafaxine).
3. <http://www.accessdata.fda.gov/drugsatfda_docs/label/2014/021992s039lbl.pdf>
4. Li Huafang. Common scale for clinical research of psychotropic drugs. Shanghai Science and Technology Education Press. 2011.

Attachments

Appendix 1: Hamilton Depression Scale -17 (HAM-D_17_)

| *Note: 5-grade scoring method with 0～4 points is used for most items. Criteria on each grade: (0) none; (1) mild; (2) moderate; (3) severe; (4) very severe. 3-grade scoring method with 0～2 points is used for few items, the grading criteria are: (0) none; (1) mild to moderate; (2) severe* | | | |
| --- | --- | --- | --- |
| **Item** | | | **Score** |
| 1 | Depressed mood (sad, hopeless, helpless, worthless) | 0=no symptom; 1=narrate only when asked;  2= express spontaneously in conversation;  3= nonverbal expression of this emotion (e.g., through expression, posture, voice and desire to cry);  4= complete expression of this emotion almost by self speech and nonverbal expression | \|_\| |
| 2 | Feeling of guilt | 0=no symptom; 1= blame yourself and feel that you have failed others;  2= Think that you have committed a crime, or think about previous mistakes or misconducts repeatedly;  3= Think of your current illness as a punishment for your own mistakes, or have evil delusion;  4= Hear accusations or condemnations and/or have threatening illusion | \|_\| |
| 3 | Suicide | 0=no symptom; 1=feel pointless in living;  2= Wish I have died or think of something related to death frequently;  3= Negative thought (suicidal thought) or suicide posture  4= Suicidal attempt (any serious attempt will be scored 4) | \|_\| |
| 4 | Trouble falling asleep | 0 = No difficulty in falling asleep;  1= Have a chief complaint of difficulty in falling asleep sometimes (e.g., still unable to fall asleep 30min after going to bed);  2= Have a chief complaint of difficulty in falling asleep every night | \|_\| |
| 5 | Shallow sleeping | 0 = No symptom;  1= Have a chief complaint of shallow sleep at midnight, frequent nightmare;  2 = Wake up at midnight (before 12:00pm) – any case of waking up will be scored 2 (not including  going to the toilet) | \|_\| |
| 6 | Early awakening | 0 = No symptom;  1= Have early awakening, but be able to fall asleep again;  2= Fail to fall asleep again after early awakening | \|_\| |
| 7 | Work and activity | 0 = No symptom;  1= Feel inadequate ability to equal to your ambition, tired, or weak during activity, work or hobby;  2= Lose interest in activity, hobby or work - direct or indirect expression of listless,  irresolute and hesitant, and hesitant (feel to have to force yourself to work or engage in activity);  3= Decreased time or efficiency of activity, daily ward activity (labor or entertainment during hospitalization) less than  3h, not including daily ward affairs for inpatients;  4= Stop work for current disease, do not engage in any activity other than daily ward affairs for inpatients, or be unable to complete daily ward affairs without other’s help | \|_\| |
| 8 | Tardiness (defined as slow thought and speech, difficulty in concentration, compromised initiative) | 0 = Normal speech and thought;  1 = Mild tardiness discovered in psychiatric examination;  2 = Obvious tardiness discovered in psychiatric examination;  3 = Difficulty in carrying out psychiatric examination;  4 = Complete stupor | \|_\| |
| 9 | Agitated | 0 = No symptom;  1 = Be agitated;  2 = Fiddle with hands, hair, etc.;  3 = Walk around, fail to sit still;  4 = Rub hands, bite nail, pull hair, bite lip | \|_\| |
| 10 | Psychic anxiety | 0 = No symptom;  1 = Subjective nervousness and irritability;  2 = Worry about minor things;  3 = Obvious anxiety revealed in expression and speech;  4 = Show fear beyond doubt | \|_\| |
| 11 | Somatic anxiety (physiological symptoms of anxiety, for example, dry mouth, shortness of breath, dyspepsia, diarrhea, abdominal colic, belching, palpitation, headache, hyperventilation, sigh, frequency of micturition, sweating) | 0 = No symptom;  1 = Mild;  2 = Moderate;  3 = Severe;  4 = Serious impact on life and activity | \|_\| |
| 12 | Gastrointestional symptom | 0 = No symptom;  1 = Decreased appetite, but being capable of self feeding without other’s encouragement, abdominal heaviness;  2 = Requiring other’s encouragemet to eat food; request or need to use laxatives or cathartic, or the drugs for gastrointestional symptoms | \|_\| |
| 13 | General symptoms | 0 = No symptom;  1= Heaviness of limbs, back or head; back pain, headache, muscle pain; systemic weakness and fatigue;  2 = Any obvious symptom | \|_\| |
| 14 | Sexual symptom (loss of sexual desire, menstrual disorder) | 0 = No symptom;.  1=Mild;  2=Severe | \|_\| |
| 15 | Hypochondriasis | 0=no symptom; 1= excessive attention to (physical health); 2=repeated consideration of health issue;  3=frequenty complaint, request for help; 4= Hypochondriac delusion | \|_\| |
| 16 | Weight decrease | 0 = No decrease of body weight;  1= decreased body weight possibly related with the existing disease;  2=positively decreased body weight (by patient);  3= Not evaluated | \|_\| |
| 17 | Insight | 0= Be aware of your own disease and appear depression;  1 = Be aware of your own disease, but attribute to too poor diet, environment issue, too busy work, viral infection or  requiring rest, etc.;  2=Completely deny disease | \|_\| |
| **Total score \|_\|_\|** | | | |

Appendix 2: Clinical global impression scale (CGI)

| **I. Severity (CGI-S)**  In combination with your overall clinical experience with this specific population, what is the severity of the patient's condition at this time?  0=Not assessed 4=Moderate  1=Normal, completely healthy 5=Obvious  2= Borderline psychosis 6=Severe  3=Mild 7=Extremely severe |
| --- |
| **II. Overall improvement (CGI-I)**  In accordance with your judgment, is this improvement completely due to the treatment of the drug? How is the condition changed compared with that at the enrollment visit?  0=Not assessed 4=No change  1=Very obvious improvement 5=Slight exacerbation  2=Obvious improvement 6=Obvious exacerbation  3=Slight improvement 7=Very obvious exacerbation |

Appendix 3: Hamilton Anxiety Scale (HAMA)

| *5-grade scoring method with 0～4 points is used for all the items. Criteria on each grade: (0) none; (1) mild; (2) moderate; (3) severe; (4) very severe.* | | |
| --- | --- | --- |
| **Item** | | **Score** |
| 1. Anxious mood | Worry, premonition that the worst will happen, fear, irritability | \|_\| |
| 2. Nervousness | Nervous, prone to tiredness, startle reaction, burst into tears easily after touched, shaking, feel uneasy and unable to be relaxed | \|_\| |
| 3. Fear | Fear of darkness, stranger, being alone, animal, taking a car or crowded places | \|_\| |
| 4. Insomnia | Difficulty in falling asleep, restless sleep, shallow sleep and fatigue after waking up, dreaminess, nightmare, night terror | \|_\| |
| 5. Memory or attention disorder | Inability to concentrate, poor memory | \|_\| |
| 6. Depressive mood | Loss of interest, lack of pleasure in previous hobbies, depression, early awakening, mild in the day and severe at night | \|_\| |
| 7. Muscle symptoms | Muscle soreness, muscle twitch, inflexible movement, myoclonic convulsion, grinding teeth in sleep, trembled voice, increased muscle tension | \|_\| |
| 8. Sensory symptoms | Tinnitus, blurred vision, coldness and hotness, weakness, tingling | \|_\| |
| 9. Cardiovascular symptom | Tachycardia, palpitation, chest pain, vascular pulsation, fainting sensation, heart beat escape | \|_\| |
| 10. Respiratory symptoms | Chest distress or tightness, feeling of suffocation, sigh, dyspnea | \|_\| |
| 11. Gastrointestinal symptoms | Dysphagia, abdominal pain and distending pain, burning sensation, abdominal distension, nausea, vomiting, bowel sound, diarrhea, decreased body weight, constipation | \|_\| |
| 12. Genitourinary symptoms | Frequency of micturition, urgency of micturition, amenorrhoea, menorrhagia, sexual apathy, premature ejaculation, hyposexuality, impotence | \|_\| |
| 13. Autonomic nervous symptoms | Dry mouth, flushing, pallor, easy sweating, vertigo, tension headache, hair rising. | \|_\| |
| 14. Behavior at interview | Restlessness, fidgety or pacing, hand shaking, frown, stiff expression, sigh or shortness of breath, pale face, swallowing from time to time | \|_\| |
| **Total score \|_\|_\|** | | |

Appendix 4: Visual Analog Scale - Pain intensity (VAS-PI)

How disturbed are you with any one of the following questions in the past week:

1. Overall pain level (VAS-PI) □No □Yes Score: |_|_|.|_|

Mark on the following scale to indicate describe the overall pain level you feel in the past week:

No pain Most serious pain

|  |
| --- |

0 point 10 points

2. Extent of headache (VAS-PI) □No □Yes Score: |_|_|.|_|

Mark on the following scale to indicate describe the extent of headache you feel in the past week:

No headache Most serious pain

|  |
| --- |

0 point 10 points

3. Extent of back pain (VAS-PI) □No □Yes Score: |_|_|.|_|

Mark on the following scale to indicate describe the extent of back pain you feel in the past week:

No back pain Most serious pain

|  |
| --- |

0 point 10 points

4. Extent of limb or joint pain (VAS-PI) □No □Yes Score: |_|_|.|_|

Mark on the following scale to indicate describe the extent of limb or joint pain you feel in the past week:

No limb or joint pain Most serious pain

|  |
| --- |

0 point 10 points

5. Extent of abdominal pain (VAS-PI) □No □Yes Score: |_|_|.|_|

Mark on the following scale to indicate describe the extent of abdominal pain you feel in the past week:

No abdominal pain Most serious pain

|  |
| --- |

0 point 10 points

6. Other pain (VAS-PI), □No □Yes, please indicate Score: |_|_|.|_|

Mark on the following scale to indicate describe the extent of other pain you feel in the past week:

No pain Most serious pain

|  |
| --- |

0 point 10 points

Appendix 5: Description of amendment clinical trial protocol

|  | Position in the original text | Text before change | Text after change | Reason(s) for Change |
| --- | --- | --- | --- | --- |
| 1 | Cover and header | Version number 1.1  Date of version August 24, 2015 | Version No. 2.0  Date of version July 1, 2016 | Protocol Amendment(s) |
| 2 | Main body |  | **Sponsor’s contact**  Shandong Luye Pharma Co Ltd  Name: Shuren GUO, M.D., Senior Director, Medical Affairs  Phone: 010-5281-9352  Cell phone: 13501029003  Fax: 010-52819299  Email: guoshuren@luye.cn  Address: No. 9 Baoyuan Road, Laishan District, Yantai, Shandong province  If you can not contact the above personnel, you can contact the following persons for all the other requirements and information on this study:  Name: Yan ZHANG, Project manager, Medical Affairs  Cell phone: 13910310185  **Contact person of investigators**  Clinical study leading unit: Peking University Sixth Hospital  Name: Hongyan ZHANG, Professor  Office phone number: 010-82013183  Cell phone: 13601237138  Fax: 010-82013183  Email: [sally_zhy@sina.com](file:///D:\linda\最终稿\2020\09\9-72\sally_zhy@sina.com)  Address: No. 51 North Huayuan Road, Haidian District, Beijing  **Contact person of statisticians**  Statistical analysis teaching and research section, the 4th Military Medical University of PLA  Name: Jielai XIA, Professor  Cell phone: 13571999716  Email: 13571999716@163.com  Address: No. 169 West Changle Road, Xincheng District, Xi’an, Shaanxi | Update of protocol template: the contact personnel of the sponsor, leading and statistical analysis unit are added |
| 3 | Main body |  | Signature page (Data Management and Statistical Analysis Unit)  I sign hereunto to indicate that I have participated in the formulation and discussion of this clinical study potocl and agreed upon the contents of the protocol. I agree to carry out the data management and statistical analysis for the clinical trial in accordance with the provisions in the protocol and all the applicable legal and regulatory requirements.  Person in charge of data management and statistical analysis  Signature_______________ ______YYYY____MM____DD  Unit name: Statistical analysis teaching and research section, the 4th Military Medical University | Update of protocol template: signature page of statistical analysis unit is added |
| 4 | Summary – course of therapy | A total of 7 weeks, including a wash-out period of 1 weeks and treatment period of 6 weeks | A total of8weeks, including a screening/wash-out period of two weeks and treatment period of 6 weeks. | As the reporting time of partial laboratory examinations is long, the screening/wash-out period is prolonged to Day -14. |
| 5 | 4.1. Test Methods | This study is comprised of two periods: screening and wash-out period (1week), double-treatment period (6 weeks). Screening eligible subjects with major depressive disorder will enter the one-week placebo wash-out period and receive placebo two tablets, once per day, for consecutive 7 days. | This study is comprised of two periods: screening and wash-out period (2 week), double-treatment period (6 weeks). The 1st period is one screening and wash-out period of 8 days (recommended) to up to 14 days, screening eligible subjects with major depressive disorder will enter the one-week placebo wash-out period and receive placebo two tablets, once per day, for consecutive 7 days. | As the reporting time of partial laboratory examinations is long, the screening/wash-out period is prolonged to Day -14. |
| 6 | 7 Study flow chart | Visit 1, -8~ -1 d | Visit 1, -14 ~ -1 d  Add: Note 1 and 7  Screening/wash-out period: Day -14~-1, all the subjects will be given the drugs for wash-out period on Day -8 and enter one 7-day wash-out period (-7 ± 1d).  7. Drugs in wash-out period: dispensed on Day -8 and used from Day -7. | As the reporting time of partial laboratory examinations is long, the screening/wash-out period is prolonged to Day -14.  Indicate the start time of drug dispensation and administration in wash-out period. |
| 7 | 7.1 Visit 1 | Visit 1— — Day -8 ~ -1  All the subjects meeting the inclusion criteria and not meeting the exclusion criteria will enter the one-week placebo wash-out period, be given the drugs for wash-out period and introduced the administration method and precautions in detail. A follow-up visit after 7 days will be scheduled. If the patient has any question on this study or abnormal condition during this period, he/she can contact the study personnel at any time. | Visit 1— —Day -14 ~ -1  All the subjects meeting the inclusion criteria and not meeting the exclusion criteria will enter the one-week placebo wash-out period. Investigators will dispense the drugs for wash-out period on Day -8 and describe the administration method and precautions in detail. Subjects will receive the drugs on the following day (Day -7) for consecutive 7 days, the acceptable time window for the wash-out period is -7 ± 1 day. A follow-up visit after 7 days will be scheduled. If the patient has any question on this study or abnormal condition during this period, he/she can contact the study personnel at any time. | As the reporting time of partial laboratory examinations is long, the screening/wash-out period is prolonged to Day -14.  Make clear the start time of drug dispensation and administration in wash-out period. |
| 8 | 8.1 Secondary variables | Change in visual analogue scale- pain intensity (VAS-PI) | Change in visual analogue scale- pain intensity (VAS-PI) score: including overall pain, headache, back pain, extremity or joint pain, abdominal pain and other pain. | The description is refined. |
| 9 | 8.2.1 Vital sign and weight, blood pressure and pulse rate measurement procedure | ……..throughout the study, the blood pressure will be measured through the same arm by the same study personnel…….. utmost effort will be made to acquire vital sign measurements at the same time point at each visit, and the vital signs should be measured by the same person. | ……..throughout the study, the blood pressure will be measured through the same arm by the same study personnel as far as possible…….. utmost effort will be made to acquire vital sign measurements at the same time point at each visit, and the vital signs should be measured by the same person as far as possible. | There is a great difficulty in clinical actual operation. |
| 10 | 8.2.3 12-lead ECG | 12-lead ECG will be performed at screening, baseline, visit 4 and 6 or premature termination, the subjects should rest in supine position for at least 5 minutes prior to each measurement. | 12-lead ECG will be performed at screening, baseline, visit 4 and 6 or premature termination, the subjects should rest for at least 5 minutes prior to each measurement. | Clarify the requirements for ECG investigation |
| 11 | 9. Adverse Event Report | Adverse event (AE) is defined as any unfavorable medical event that occurs in the subject who receives study drug in clinical trial, which does not necessarily have a causal relationship with the drug. In this study, record of adverse event is mainly the subjective symptoms and physicochemical examination after administration. | Adverse event (AE) is defined as any unfavorable medical event that occurs in the subject who receives study drug in clinical trial, which does not necessarily have a causal relationship with the drug. In this study, adverse event (serious adverse event and non-serious adverse event) from the signature of informed consent form to the last visit will be recorded. | Clarify the time of adverse event record |
| 12 | 12. Quality control and assurance (1) | The study drug will be maintained by the person in charge of drug management, locked in a specially assigned counter and kept at room temperature. The residual drugs will be stored separately and registered for the amount, and returned to the sponsor centrally at the end of the study. | The study drug will be maintained by the person in charge of drug management, locked in a specially assigned counter and kept at normal atmospheric temperature. The residual drugs will be stored separately and registered for the amount, and returned to the sponsor centrally at the end of the study. | Unify the wording |
| 13 | Appendix 5 Visual Analog Scale – Pain Intensity (VAS-PI) | Sample Table :  There is a 10 cm horizontal line below. One end of the horizontal line is 0, indicating no pain, and the other end is 10, indicating severe pain, and the middle represents different degrees of pain. Please ask the subject to mark the horizontal line according to their own feelings to indicate the intensity of the pain.  No pain Unbearable severe pain   \|  \| \| --- \|   0 10 | **How disturbed are you with any one of the following questions in the past week:**  **1. Overall pain level (VAS-PI) □No □Yes Score: \|_\|_\|.\|_\|**  Mark on the following scale to indicate describe the overall pain level you feel in the past week:  No pain Most serious pain   \|  \| \| --- \|   0 10  **2. Extent of headache (VAS-PI) □No □Yes Score: \|_\|_\|.\|_\|**  Mark on the following scale to indicate describe the extent of headache you feel in the past week:  No headache Most serious pain   \|  \| \| --- \|   0 10  3. **Extent of back pain (VAS-PI) □No □Yes Score: \|_\|_\|.\|_\|**  Mark on the following scale to indicate describe the extent of back pain you feel in the past week:  No back pain Most serious pain   \|  \| \| --- \|   0 10  4. **Extent of limb or joint pain (VAS-PI) □No □Yes Score: \|_\|_\|.\|_\|**  Mark on the following scale to indicate describe the extent of limb or joint pain you feel in the past week:  No limb or joint pain Most serious pain   \|  \| \| --- \|   0 10  5. **Extent of abdominal pain (VAS-PI) □No □Yes Score: \|_\|_\|.\|_\|**  Mark on the following scale to indicate describe the extent of abdominal pain you feel in the past week:  No abdominal pain Most serious pain   \|  \| \| --- \|   0 10  **6. Other pain (VAS-PI) □No □Yes, please indicate Score: \|_\|_\|.\|_\|**  Mark on the following scale to indicate describe the extent of other pain you feel in the past week:  No pain Most serious pain   \|  \| \| --- \|   0 10 | The content is refined. |
